# A comprehensive evaluation of non-vascular prepontine cistern anatomy influencing trigeminal nerve vulnerability using machine learning-based morphometric analysis

**DOI:** 10.3389/fmed.2026.1745815

**Published:** 2026-03-03

**Authors:** Akçay Övünç Karadaş, Gokalp Tulum, Ömer Karadaş, Muhammet İkbal Işık, Ferhat Cüce, Onur Osman, Zehra Şimşek, Necibe Sare Mert, Niray Baş, Berza Özcan, Tuba Baldan Ağaoğlu

**Affiliations:** 1 Private Clinic, Ankara, Türkiye; 2 Department of Electrical and Electronics Engineering, Istanbul Topkapi University, Istanbul, Türkiye; 3 Department of Neurology, Gulhane Training and Research Hospital, Health Science University, Ankara, Türkiye; 4 Department of Radiology, Gulhane Training and Research Hospital, Health Science University, Ankara, Türkiye; 5 Department of Software Engineering, Istanbul Topkapi University, Istanbul, Türkiye; 6 Department of Information Systems Engineering, Istanbul Topkapi University, Istanbul, Türkiye

**Keywords:** LIME, machine learning, Meckel cave, morphometric MRI, neurovascular conflict, prepontine cistern, SHAP (SHapley Additive exPlanations), trigeminal neuralgia

## Abstract

**Background:**

Trigeminal neuralgia (TN) is a severe neuropathic pain disorder traditionally attributed to neurovascular compression. However, emerging evidence suggests that non-vascular anatomical variations of the prepontine cistern may significantly contribute to disease susceptibility.

**Objective:**

To quantify non-vascular morphometric features of the trigeminal nerve and adjacent cistern and evaluate their discriminative value using a leakage-free, machine-learning-based MRI pipeline.

**Methods:**

We retrospectively analyzed 131 participants (71 with idiopathic TN (iTN) and 60 controls) who were imaged with temporal MRI. Two neuroradiologists independently assessed the neurovascular conflict status, achieving inter-rater agreement of 97% (*κ* = 0.91). Measured parameters included trigeminal nerve thickness (root and porus trigeminus level), Meckel cave area (axial and coronal plane) and height (sagittal plane), cisternal length (Mean), cisternopontine angle, sagittal angle, and trigeminoclival angle. Model selection employed nested, paired splits across 20 outer repetitions with Optuna-based tuning; average precision (PR-AUC) was the optimization target. Six classifier families (Random Forest, SVM, MLP, XGBoost, KNN, Bagging) were evaluated; SHAP and LIME were applied *post-hoc* for interpretability.

**Results:**

TN showed thinner nerve diameters (particularly at the porus), larger Meckel cave areas (axial and coronal) and height, smaller sagittal angles, and shorter cisternal length; several of these differences remained significant after multiple-comparison control (e.g., porus diameters and Meckel cave areas, Holm-adjusted *p* < 0.01; sagittal angle, Holm *p* = 0.0092). On held-out test sets, discrimination was consistently high: for SVM, PR-AUC was 86.16 ± 4.39% and ROC-AUC was 87.40 ± 4.52%; the other models clustered closely around ROC-AUC (≈0.85–0.87). Friedman testing demonstrated a global difference on *F*_1_ across models; *post-hoc* Wilcoxon–Holm confirmed that only Random Forest exceeded KNN, while RF, SVM, and XGBoost did not differ pairwise on *F*_1_ or ROC AUC. SHAP/LIME prioritized porus-level diameters and Meckel cave measures as leading contributors, aligning with groupwise morphometric shifts.

**Conclusion:**

Non-vascular morphometric variation in the prepontine cistern, particularly at the porus level nerve caliber, Meckel cave size, and sagittal angle, contributes to TN pathophysiology. An AI-assisted, leakage-free morphometry pipeline yields reproducible and interpretable discrimination, supporting the integration of vascular and non-vascular anatomy into diagnostic and treatment planning workflows.

## 1 Introduction

Trigeminal neuralgia (TN) is a chronic neuropathic pain disorder, one of the most frequent and painful diseases. TN is presented as recurrent episodes of sudden, short pain resembling electric shocks involving the facial dermatome of the trigeminal nerve. Although the diagnosis of TN depends on symptoms and patient history, the treatment algorithm is determined according to the MRI result.

According to the International Classification of Headache Disorders (ICHD-3) guidelines, TN is classified as classical, secondary, or idiopathic based on underlying mechanisms. Classical TN (cTN) occurs in the presence of severe (Grade 2 and Grade 3) neurovascular conflict (NVC) with visual morphological changes in the trigeminal nerve; secondary TN due to an underlying lesion or disease instead of vessel contact; and idiopathic TN (iTN) with no identifiable etiology or mild (Grade 1) NVC without any morphological changes of nerve. This classification system, updated in 2018, provides a more comprehensive understanding of the different forms of TN and their underlying causes (1).

The primary imaging method for defining etiology is magnetic resonance imaging (MRI), which provides high resolution of the posterior fossa. Balanced steady-state free precession (bSSFP) sequences, such as constructive interference steady state (CISS) and fast imaging employing steady-state acquisition (FIESTA), with a thin section to the prepontine cistern, can reveal NVC, nerve atrophy, prepontine cistern anatomy, and other anatomy of Meckel cave and petrous bone (2, 3).

The most common form is cTN. The prevailing theory regarding cTN is that NVC induces localized demyelination of the nerve, which subsequently triggers the abnormal generation of nerve impulses. NVC has been proven histopathologically to play a role in the demyelination of the trigeminal nerve (4). This understanding highlights the importance of addressing NVC to effectively manage cTN (5). However, NVC does not always seem to be sufficient to cause demyelination of the nerve, nor does NVC appear to be essential for TN (6). NVC has been reported in 48.9% of the contralateral asymptomatic side of TN (7). The occurrence of NVC in healthy populations suggests that vascular contact is insufficient to explain the cause of TN (8) fully. Considering the various predisposing factors is crucial for understanding the development of TN and ensuring its effective treatment (9). The role of NVC may act as a trigger in individuals already predisposed to developing TN.

Several anatomical structures of the posterior fossa have been studied comparatively between patients with TN and the general population. The morphometric differences of the trigeminal pontine angle, the length and thickness of the trigeminal nerve, the distance between two trigeminal nerves, the area of the pons, posterior fossa, cerebellopontine cistern and prepontine cistern, height and width of Meckel cave, petrous bone angle with trigeminal nerve, could be involved in the etiology of nerve demyelination and clinic (10–16). In MRI, radiologists can visually assess morphometric structures and obtain precise numerical measurements, enhancing the quality of diagnostic evaluations. Constructing a diagnostic criteria set based on these non-vascular anatomical parameters is also possible. A major difficulty of the conventional visual evaluation of the prepontine cistern morphometry is that there are no set norms for the anatomical measurements and the resulting variability across different observers in their assessments is hard to control.

Recent advances in artificial intelligence have increasingly been leveraged across healthcare to support more standardized, reproducible, and scalable clinical decision support. In medical imaging, AI-based computer-aided diagnostic systems have been proposed to reduce reader variability and assist clinical interpretation; for example, transfer learning has been used to develop automated grading systems for knee osteoarthritis from radiographs to support early and more consistent assessment (17). Beyond imaging, AI-driven data mining and multivariate feature extraction approaches have also been applied in biomedical research settings, such as analyzing genomic sequence similarity and motif patterns to investigate disease susceptibility and related biological links (18).

The research was designed to thoroughly explore prepontine cistern morphometry through AI-assisted MRI analysis considering non-vascular anatomical variations as one of the etiological factors of iTN.

## 2 Materials and methods

### 2.1 Patient

We conducted a retrospective study of patients clinically diagnosed with TN who underwent temporal bone MRI between January 2019 and January 2025. We included 71 iTN patients with unilateral pain. We accepted the symptomatic side as the patient group. Operationally, classical TN and secondary TN were excluded; thus, the iTN cohort comprised patients without an identifiable secondary cause and without severe NVC-related trigeminal morphological changes (i.e., not meeting cTN criteria). Controls were defined as pain-free participants with Grade 0 and/or Grade 1 NVC on temporal bone MRI. Additionally, we created a control group free from facial pain, including 60 participants with Grade 0 NVC and Grade 1 NVC with temporal bone MRIs. For controls, both trigeminal nerve sides were included as asymptomatic observations (two observations per participant), yielding *n* (Class-0) = 120 from 60 controls. The total number of participants in the study across the two groups was 131. The exclusion criteria were: (i) poor diagnostic quality of MRI, (ii) cTN to avoid the possibility of demyelination due to vascular factors, secondary TN patients, and (iii) TN patients who had received medical or interventional treatment.

### 2.2 Imaging parameters

The MRIs were performed on a Philips 3T imaging system with a dedicated head coil. The temporal bone MR imaging protocol included a 20 cm × 20 cm field of view and a 512 × 256 matrix size. The axial fast spin-echo T2-weighted imaging parameters were as follows: TR: 4,000 ms, TE: 98 ms, with 2 mm slice thickness with 1 mm interslice gap. The axial spin-echo T1-weighted imaging parameters were as follows: TR: 400 ms, TE: 9 ms, with 2 mm slice thickness with 1 mm interslice gap. The key sequence for morphometry was the balanced fast field echo (BFFE): TE 5.5 ms, TR 11 ms, flip angle 45°, ETL 1, slice thickness 1 mm, voxel size 1 × 1 × 1 mm^3^.

### 2.3 Measurement of anatomical morphometry

Two independent radiologists (MI and FC), experienced in neuroradiology with 5 and 15 years of experience, and blinded to the clinical findings, assessed morphometric analysis and segmented the images in both groups using ManSeg (v.3.0) (19).

**Figure 1:**
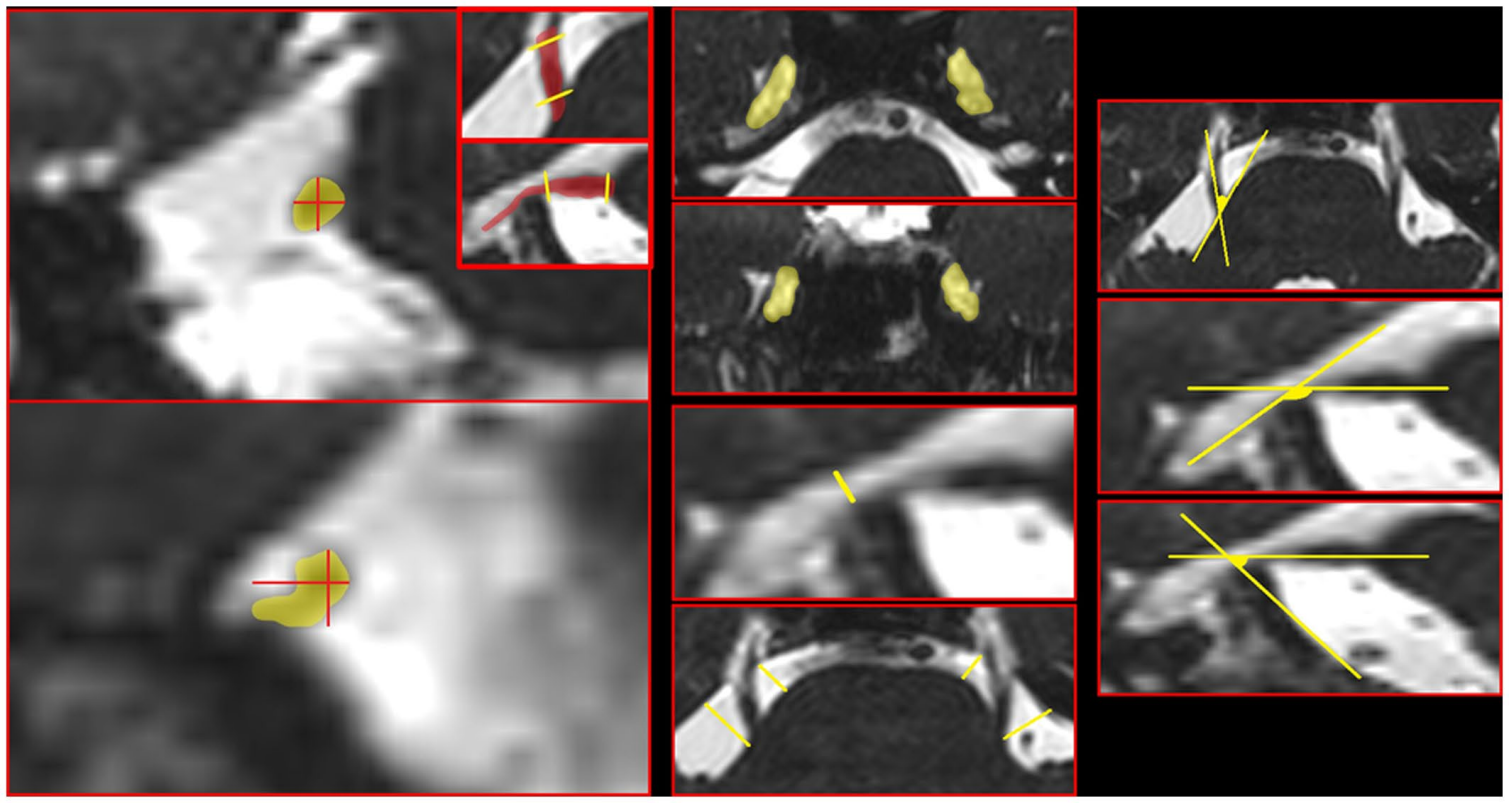
Measurement of prepontine anatomical parameters. All measurements were made by reconstructing images parallel to the long axis of the cisternal segment of the trigeminal nerve. From top to bottom and left to right, the measurements are as follows: nerve diameter (root), nerve diameter (porus trigeminus), Meckel cave area (axial plane), Meckel cave area (coronal plane), Meckel cave height, cisternal length (Mean), cisternopontine angle, sagittal angle, and trigeminoclival angle.

To assess anatomical structures, the BFFE sequence was used. All morphometric measurements were performed on the BFFE sequence after reconstructing images parallel to the long axis of the cisternal segment of the trigeminal nerve (Figure 1). The measured parameters included trigeminal nerve transverse and vertical diameters at the root and porus trigeminus levels (mm); Meckel’s cave area on the axial and coronal planes (cm^2^); Meckel’s cave height on the sagittal plane (mm); prepontine cistern length computed as the mean of perpendicular distances (mm); and angular measurements including the cisternopontine angle, sagittal angle, and trigeminoclival angle (degrees).

Trigeminal nerve thickness was measured at the root and porus trigeminus levels in both the transverse and vertical planes. Meckel cave area was measured in two dimensions using the widest slices in the axial and coronal planes. The height of Meckel cave was measured from the level of the cave mouth in the sagittal plane. The prepontine cistern length was calculated by averaging the perpendicular lengths drawn from the lateral contour of the pons to both root and porus trigeminus medial side. The angle between the long axis of the nerve and the pons was defined as the cisternopontine angle. In cisternopontine angle measurement, one end of the angle was defined to be tangent to the edge of the pons, and the other end was standardized by extending it from the middle section of the trigeminal nerve towards Meckel’s cave. The sagittal angle measurement was performed between the long axis of the nerve and the petrous bone upper contour on the sagittal plane.

Additionally, the trigeminoclival angle was also measured between the nerve and the clival ridge on the sagittal plane (Figure 1). All measurements were repeated at least twice, and the final values were obtained by averaging the measurements of two radiologists. The interrater reliability between the two observers showed agreement in identifying NVC (97% agreement, kappa coefficient 0.91). Inter-rater reliability for continuous morphometrics was not estimated as ICC; measurement values were averaged across two readers.

### 2.4 Methods

We used Optuna (20) to construct a leakage-free nested model-selection workflow. During each repetition, StratifiedShuffleSplit generated a test set that was not seen and consisted of 20% of the data which maintains class proportions while shuffling indices. After holding out an outer test set of 20%, the remaining 80% was split into inner training and validation sets, yielding an overall composition of approximately 65% training, 15% validation, and 20% test (i.e., 81.25% training and 18.75% validation within the train + validation subset). All data preprocessing and feature filtering were performed within the estimator pipeline and fitted only once on the inner training fold to prevent information leakage. Continuous features were standardized by RobustScaler, which is based on the median and interquartile range and is not affected by outliers. Univariate filtering used SelectKBest with the ANOVA *F*-statistic (21), keeping top-*k* features. The number of features selected, *k*, was defined as a hyperparameter that could be tuned, with a range of 5 to 12, and was modified according to the dimensionality of the data after preprocessing. No imputation was necessary as there were no missing values in the analysis dataset across the features that were used. The categorical feature (Sex) was one-hot encoded and sent through without scaling; only the continuous features were scaled.

Hyperparameters were optimized using Optuna’s Tree-structured Parzen Estimator (TPE) (22), a Bayesian search strategy that models the relationship between performance and parameters, along with a MedianPruner for early stopping after a 10-step warm-up when interim scores lagged behind the running median. For each classifier, the search was run for 150 trials. Searches were run on the same inner and outer splits for six classifiers: Random Forest, SVM, MLP, XGBoost, KNN, and Bagging. For Random Forest, we tuned the number of trees, maximum depth, minimum samples for splits and leaves, the feature subsampling policy, the impurity criterion, and the bootstrap option. Class imbalance was handled with balanced class weights, which set weights inversely proportional to class frequency. For SVM, we tuned the kernel (linear, rbf, or polynomial), the penalty parameter C, and, when relevant, the gamma parameter (scale, auto, or a positive value) and the polynomial degree; probabilistic outputs were enabled to compute ranking-based metrics such as average precision and ROC-AUC, and we used balanced class weights to mitigate imbalance. For MLP, we tuned the hidden-layer topology, activation function, L2 penalty (alpha), solver (adam or lbfgs), maximum iterations, and, when adam was selected, the learning-rate policy, initial step size, beta-1, beta-2, early stopping, and batch size. For XGBoost, the search covered the number of estimators, depth, learning rate, subsampling and column-sampling ratios, minimum child weight, gamma, and the regularization terms (reg_alpha and reg_lambda); the positive-class scaling factor was adjusted in proportion to the empirical imbalance, a histogram-based tree builder was used for efficiency, log-loss was used as the optimization metric, and parallel workers were employed. For KNN, we tuned the number of neighbors, the distance metric, and Minkowski order, the weighting scheme, and the leaf size. For Bagging, we selected a base learner, either a class-balanced decision tree with tuned depth and split/leaf thresholds or a KNN with tuned neighbors and weights. We then optimized the ensemble size, sample and feature fractions, and bootstrap options. Random seeds were fixed per repetition for the outer and inner splits, each model, and the sampler to ensure reproducibility. The same outer and inner splits were reused across all models within each repetition to ensure a fair, paired comparison.

During tuning, models were wrapped in a standard scikit-learn pipeline (23), in which RobustScaler first scales all continuous features, SelectKBest then retains the top-*k* features based on the ANOVA *F*-statistic, and the chosen classifier is finally trained on the resulting subset. Each Optuna trial was trained on the inner training fold and evaluated on the inner validation fold. We recorded *F*_1_ (binary, with the predefined positive label), average precision, which is the area under the precision-recall curve (24), and ROC-AUC, and we maximized average precision on the validation set as the optimization objective. Predicted labels were obtained by thresholding class probabilities at 0.5. Class probabilities were used to compute ranking-based metrics (average precision and ROC-AUC), with the Class-1 index aligned to each classifier’s class ordering.

After optimization, the best hyperparameters were consolidated, and the winning configuration was refit on the combined training and validation data with the selected *k* and the fixed preprocessor. We stored the selected-feature mask and names before and after preprocessing together with the vectors of ANOVA *F*-scores and *p*-values returned by SelectKBest. The final performance on the untouched test set included *F*_1_-score, average precision, and ROC-AUC, along with the confusion matrix, the ROC curve (false-positive rate and true-positive rate), and the precision-recall curve (precision and recall).

**Figure 2:**
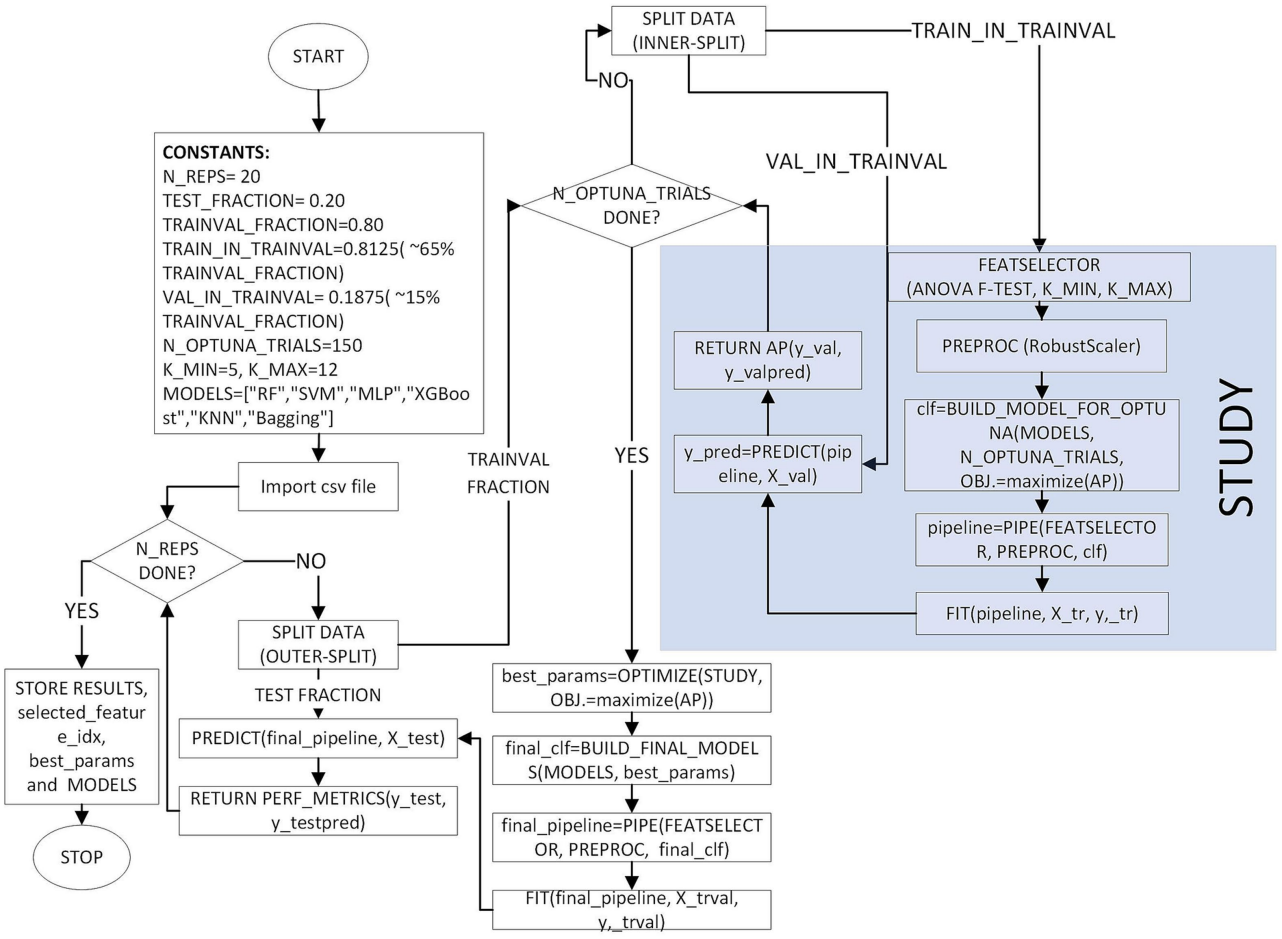
Flowchart of the nested model-selection and evaluation procedure.

During the 20 outer repetitions, the performance was expressed as mean ± standard deviation. The comparison of model families over repetitions was done by a Friedman test followed by Wilcoxon signed-rank tests with Holm correction (for details see Results) (25). Supplementary Table S2 lists the full hyperparameter search spaces, including the bounds, steps, and priors. Figure 2 illustrates a complete end-to-end flowchart of the methodology starting from the stratified splits through Optuna-based tuning to final testing. The entire source code that was used in this research has been published on GitHub at https://github.com/DrGokalpTulum/iTN-study.git.

In order to provide understandable insights along with the aggregate performance metrics, we employed *post-hoc* explainable AI (XAI) methods, SHAP (SHapley Additive exPlanationss) (26) and LIME (Local Interpretable Model-agnostic Explanations) (27, 28), to find out which morphometric features had the greatest impact on the predictions of the model from both a global (feature importance patterns) and a local (case-level explanations) perspective, and to examine the degree of consistency with respect to these attributions across different models and repetitions. The Results section includes a concise summary of the findings, while all figures and detailed outputs can be found in the Supplementary material.

## 3 Results

### 3.1 Descriptive statistics and exploratory data analysis (EDA)

**Figure 3:**
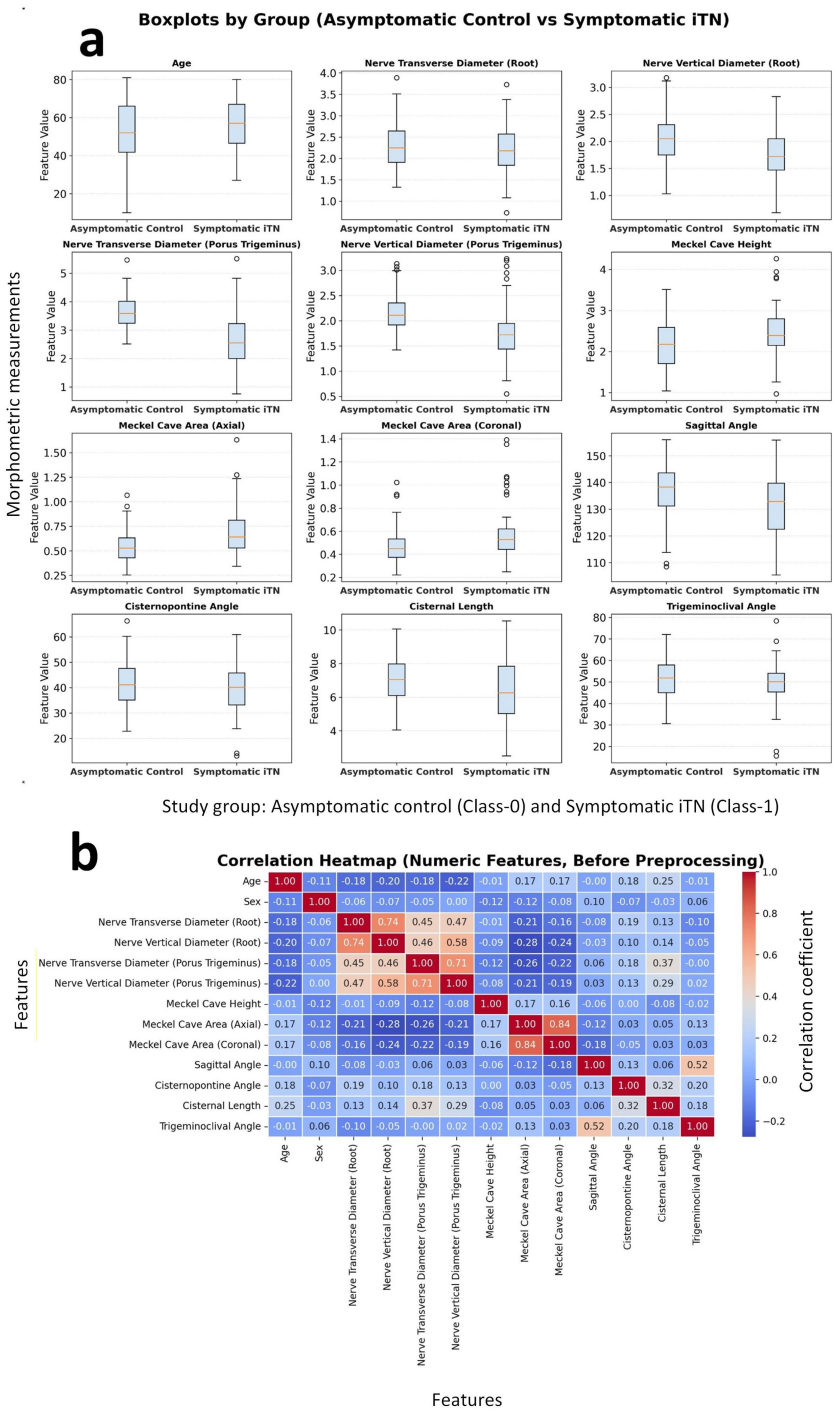
**(a)** Boxplots of numerical features comparing the asymptomatic control and symptomatic iTN groups. **(b)** Correlation heatmap of features.

We deliver a systematic overview of the various numerical variable distributions for different classes and their relationships with each other. To be precise, “Class-0” refers to the asymptomatic/control group while “Class-1” refers to the symptomatic/case group. The boxplots for all numerical features are shown in Figure 3a, which help to understand the central tendency and variation of each variable by class. There are significant differences in the positions of the nerve diameters among the classes, especially at the root and porus trigeminus locations where both the transverse and vertical diameters in Class 1 have lower central values. On the other hand, the variables like cisternal length and age have distributions that significantly overlap with each other indicating that their class-dependent effects are of small to moderate size.

Linear relationships among variables are summarized in the correlation heatmap in Figure 3b. Higher correlations appear among measurements that represent the same anatomical structures: the Meckel cave area (axial-coronal) correlates at approximately 0.84; the root-level transverse-vertical diameters at around 0.74; and the sagittal and trigeminoclival angles at about 0.52. Overall, most correlations fall within the low-to-moderate range, indicating a limited risk of multicollinearity; however, caution is warranted when jointly modeling area measures and root diameters.

The visual findings are quantitatively supported by the class-based statistical comparisons summarized in Table 1. For each variable, the table reports group sample sizes, measures of central tendency, mean differences (Δ Mean) and their direction, parametric (*t*-test, two-tailed) and nonparametric (Mann–Whitney *U*) *p*-values, homogeneity of variances (Levene’s test), effect sizes (Cohen’s *d*, Cliff’s *δ*, *ϕ*), and multiple-comparison corrections (FDR-BH and Holm). A total of 13 variables were evaluated; under Holm-adjusted *p* < 0.01, significant morphometrics were porus trigeminus transverse and vertical diameters, Meckel’s cave area (axial and coronal), root-level vertical diameter, and sagittal angle, whereas under FDR-BH <0.01, Meckel’s cave height was additionally significant (Table 1; Supplementary Table S1). The largest effect sizes were observed for the porus trigeminus transverse (*d* ≈ −1.31, Class-1 < Class-0) and vertical diameters (*d* ≈ −0.89, Class-1 < Class-0), followed by the Meckel’s cave area (axial) (*d* ≈ 0.75, Class-1 > Class-0), root-level vertical diameter (*d* ≈ −0.72, Class-1 < Class-0), Meckel’s cave area (coronal) (*d* ≈ 0.59, Class-1 > Class-0), and sagittal angle (*d* ≈ −0.52, Class-1 < Class-0). This pattern is consistent with the positional differences illustrated in Figure 3a. Full statistical metrics (all *p*-values, effect sizes, and multiple-comparison results) are provided in Supplementary Table S1. Across group-level comparisons (Table 1; Supplementary Table S1), the symptomatic iTN group shows thinner trigeminal nerve diameters, most prominently at the porus trigeminus, together with larger Meckel’s cave area measures. The symptomatic group also shows a smaller sagittal angle and shorter cisternal length. Under Holm-adjusted *p* < 0.01, the most robust differences are concentrated in porus-level diameters and Meckel’s cave areas, with sagittal angle (and root-level vertical diameter) also remaining significant, whereas Meckel’s cave height and cisternal length do not meet Holm *p* < 0.01.

**Table 1:** Summary of feature-level group comparisons (asymptomatic control = Class-0; symptomatic iTN = Class-1).

Feature	*n* (Class-1)	*n* (Class-0)	Mean (Class-1)	Mean (Class-0)	Δ Mean Class-1 − Class-0	*t*-test *p*	MWU *p*	FDR-BH *p*	Holm *p*	Cohen’s *d*	Cliff’s *δ*
Nerve transverse diameter (porus trigeminus)	71	120	2.652	3.616	−0.964	3.05 × 10^−13^	1.82 × 10^−14^	2.37 × 10^−13^	2.37 × 10^−13^	−1.309	−0.664
Nerve vertical diameter (porus trigeminus)	71	120	1.766	2.176	−0.41	1.04 × 10^−7^	2.13 × 10^−10^	1.38 × 10^−9^	2.56 × 10^−9^	−0.891	−0.551
Meckel cave area (axial)	71	120	0.701	0.548	0.153	0.0000056	2.2 × 10^−6^	9.52 × 10^−6^	2.42 × 10^−5^	0.746	0.41
Nerve vertical diameter (root)	71	120	1.746	2.062	−0.316	0.00000356	1.28 × 10^−5^	0.0000416	0.000128	−0.723	−0.378
Meckel cave area (coronal)	71	120	0.574	0.465	0.108	0.000304	0.000102	0.000266	0.00092	0.593	0.337
Sagittal angle	71	120	131.854	137.175	−5.321	0.001	0.0011	0.0025	0.0092	−0.517	−0.282
Meckel cave height	71	120	2.437	2.154	0.283	0.0014	0.0022	0.0042	0.0157	0.491	0.265
Cisternal length	71	120	6.417	7.046	−0.629	0.0161	0.0065	0.0106	0.0391	−0.384	−0.236
Trigeminoclival angle	71	120	49.214	51.417	−2.203	0.13	0.1488	0.1898	0.7442	−0.23	−0.125
Age	71	120	56.127	51.75	4.377	0.0555	0.1568	0.1898	0.7442	0.279	0.123
Nerve transverse diameter (root)	71	120	2.175	2.328	−0.153	0.0733	0.1606	0.1898	0.7442	−0.272	−0.122
Cisternopontine angle	71	120	39.717	41.288	−1.571	0.2717	0.2847	0.3084	0.7442	−0.168	−0.093
Sex	71	120						0.6413	0.7442		

For each variable, the table reports class-specific sample sizes, means, the mean difference (Δ Mean = Class-1 − Class-0; negative values indicate lower central tendency in the symptomatic group), two-tailed *t*-test and Mann–Whitney U (MWU) *p*-values, multiple-comparison–adjusted *p*-values (FDR-Benjamini–Hochberg and Holm), and effect sizes (Cohen’s *d* and Cliff’s *δ*; sign aligns with Δ Mean). Continuous variables are analyzed using both parametric and nonparametric tests, as listed. n denotes nerve-side observations.

### 3.2 Comparative performance of machine learning classifiers

**Figure 4:**
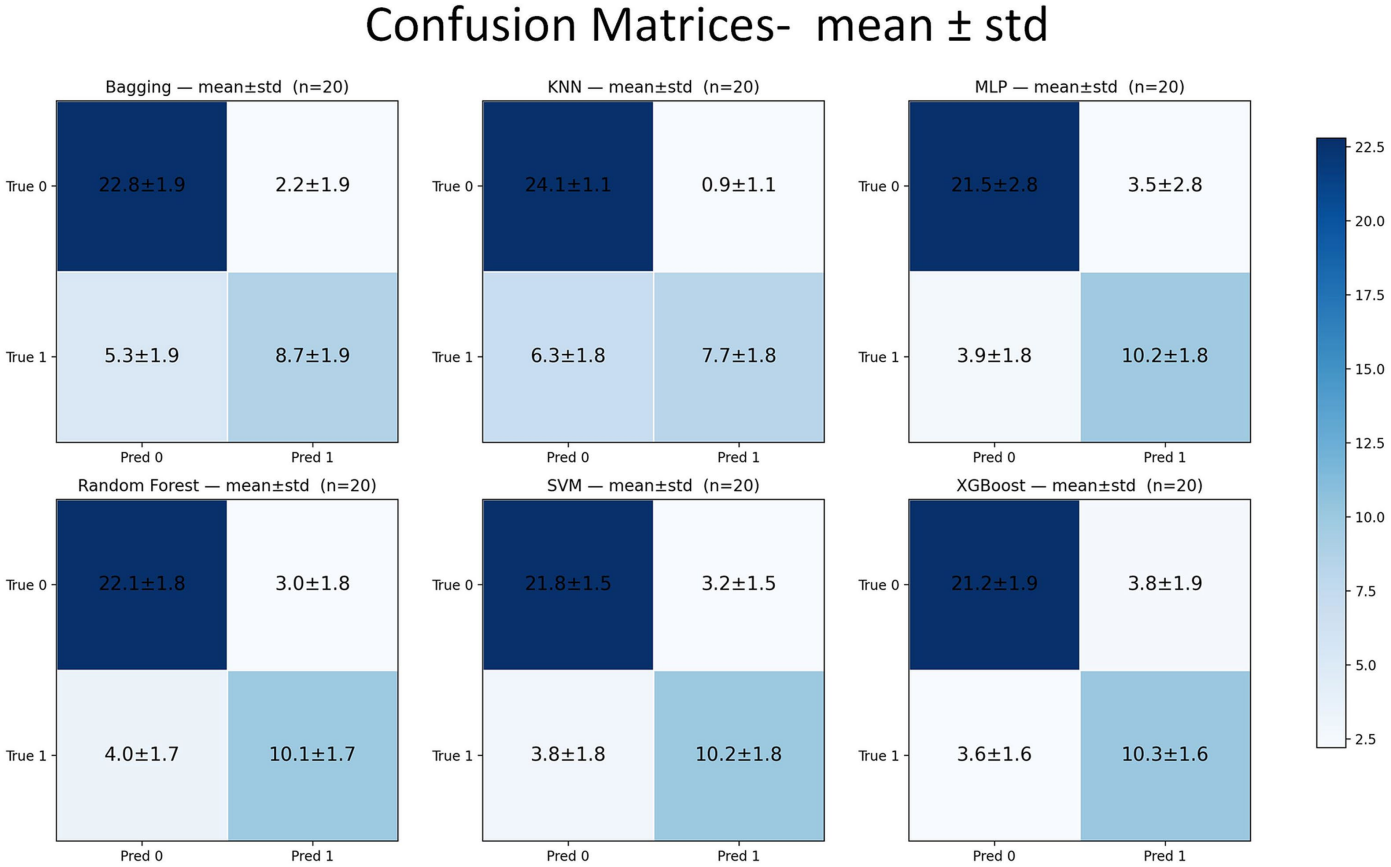
Confusion matrices averaged over 20 outer repetitions (mean ± std).

**Figure 5:**
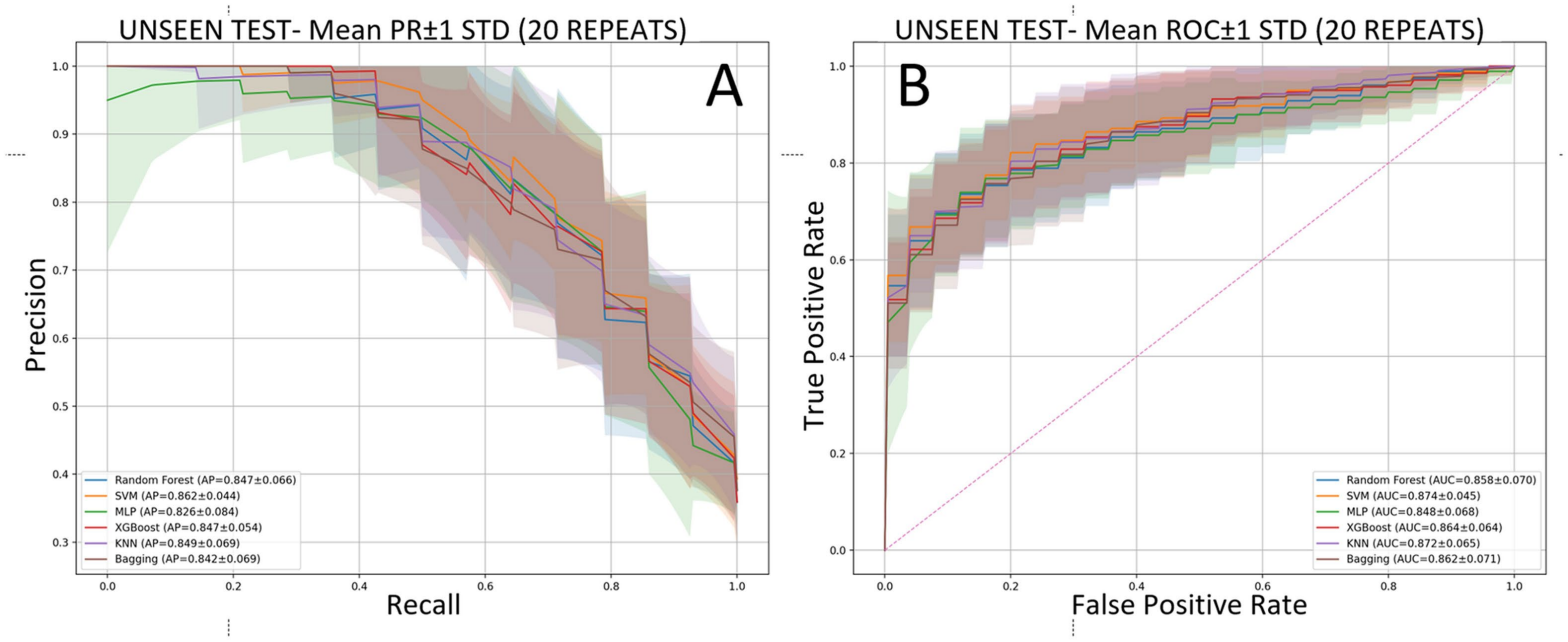
Threshold-independent discrimination on the held-out test sets across 20 repetitions. **(A)** Precision-recall curves with ±1 standard-deviation bands. **(B)** ROC curves with ±1 standard-deviation bands.

Across 20 independent outer repetitions, all six classifiers achieved good discrimination on the held-out test sets, with relatively narrow confidence bands on both precision-recall (PR) and ROC curves. The support vector machine (SVM) provided the strongest threshold-independent ranking performance, reaching a PR-AUC of 86.16 ± 4.39% (95% CI, 84.10–88.21) and a ROC-AUC of 87.40 ± 4.52% (85.28–89.52). The remaining models followed closely on ROC-AUC, with KNN at 87.23 ± 6.53%, XGBoost at 86.41 ± 6.41%, Bagging at 86.22 ± 7.05%, Random Forest (RF) at 85.84 ± 6.99%, and MLP at 84.81 ± 6.82%, all with largely overlapping ±1 SD bands on the curves. In contrast, RF led the threshold-dependent summaries: *F*_1_ was 74.22 ± 9.03% (70.00–78.45), accompanied by the highest accuracy [82.31 ± 5.99% (79.50–85.11)], G-mean [79.20 ± 7.39% (75.74–82.66)], and MCC [61.68 ± 13.18% (55.52–67.85)]. KNN exhibited the most conservative behavior, maximizing specificity [96.20 ± 4.20% (94.23–98.17)] and precision [89.67 ± 10.27% (84.87–94.48)] at the expense of recall [54.64 ± 12.54% (48.77–60.51)]. MLP and XGBoost produced the strongest recalls, 72.50 ± 13.17% (66.34–78.66) and 73.93 ± 11.65% (68.48–79.38), respectively, with SVM close behind at 72.86 ± 12.61% (66.96–78.76). These tendencies at the 0.5 probability threshold are consistent with the averaged confusion matrices (Figure 4): KNN minimizes false positives and yields the largest true negative counts (high specificity/precision) at the expense of recall, whereas MLP and XGBoost increase true positives (higher recall) with more false positives; RF and SVM maintain comparatively balanced error profiles across cells (Table 2 and Figures 4, 5A,B).

**Table 2:** Test performance of six classifiers across 20 independent outer repetitions [mean ± std (95% CI), %].

[Mean ± std (95% CI low–95% CI high), %]	Accuracy	*F*_1_	G-mean	MCC	Precision	Recall	Specificity	PR-AUC	AUC
Bagging	80.77 ± 5.55 (78.17–83.37)	69.38 ± 9.90 (64.74–74.01)	74.65 ± 7.99 (70.91–78.39)	58.06 ± 12.91 (52.02–64.10)	82.20 ± 13.45 (75.90–88.49)	62.14 ± 13.73 (55.72–68.57)	91.20 ± 7.52 (87.68–94.72)	84.21 ± 6.90 (80.98–87.44)	86.22 ± 7.05 (82.92–89.52)
KNN	81.28 ± 5.20 (78.85–83.72)	67.06 ± 10.81 (62.00–72.12)	72.01 ± 8.59 (67.99–76.03)	59.04 ± 12.39 (53.24–64.84)	89.67 ± 10.27 (84.87–94.48)	54.64 ± 12.54 (48.77–60.51)	96.20 ± 4.20 (94.23–98.17)	84.86 ± 6.91 (81.62–88.09)	87.23 ± 6.53 (84.17–90.29)
MLP	81.15 ± 6.56 (78.08–84.23)	73.27 ± 8.95 (69.09–77.46)	78.21 ± 7.68 (74.62–81.80)	60.26 ± 12.54 (54.39–66.13)	77.18 ± 13.01 (71.10–83.27)	72.50 ± 13.17 (66.34–78.66)	86.00 ± 11.13 (80.79–91.21)	82.60 ± 8.39 (78.67–86.53)	84.81 ± 6.82 (81.62–88.00)
Random Forest	82.31 ± 5.99 (79.50–85.11)	74.22 ± 9.03 (70.00–78.45)	79.20 ± 7.39 (75.74–82.66)	61.68 ± 13.18 (55.52–67.85)	78.57 ± 11.29 (73.29–83.85)	71.79 ± 12.15 (66.10–77.47)	88.20 ± 7.05 (84.90–91.50)	84.71 ± 6.63 (81.60–87.81)	85.84 ± 6.99 (82.57–89.11)
SVM	82.05 ± 3.43 (80.45–83.66)	73.96 ± 7.12 (70.62–77.29)	79.18 ± 6.24 (76.25–82.10)	61.41 ± 7.68 (57.81–65.00)	77.57 ± 8.65 (73.52–81.62)	72.86 ± 12.61 (66.96–78.76)	87.20 ± 5.89 (84.44–89.96)	86.16 ± 4.39 (84.10–88.21)	87.40 ± 4.52 (85.28–89.52)
XGBoost	80.90 ± 5.55 (78.30–83.49)	73.38 ± 8.09 (69.59–77.17)	78.80 ± 6.51 (75.75–81.85)	59.19 ± 12.06 (53.54–64.83)	74.05 ± 9.44 (69.63–78.47)	73.93 ± 11.65 (68.48–79.38)	84.80 ± 7.41 (81.33–88.27)	84.69 ± 5.42 (82.15–87.22)	86.41 ± 6.41 (83.42–89.41)

Rows list models; columns report accuracy, *F*_1_, G-mean, MCC, precision, recall (sensitivity), specificity, PR-AUC, and AUC.

Friedman’s test on *F*_1_-scored across 20 outer repetitions per each pair of classifiers showed that there are ranked differences among classifiers which are significant at an overall level (*χ*^2^ = 16.47, df = 5, *p* = 0.0056). The Friedman tests for other metrics (sensitivity, specificity, precision, recall, G-mean, MCC, and accuracy) are given in Supplementary Table S3, where it can be seen that sensitivity (*p* = 2.05 × 10^−9^), specificity (*p* = 1.33 × 10^−6^), precision (*p* = 9.15 × 10^−5^), recall (*p* = 2.05 × 10^−9^), and G-mean (*p* = 0.00027) all received significant differences across classifiers, while performance measures MCC (*p* = 0.64) and accuracy (*p* = 0.92) did not differentiate among classifiers. *Post-hoc* Wilcoxon signed-rank tests with Holm correction (Table 3) showed that Random Forest (RF) attained a significantly higher mean *F*_1_ than KNN (Δ*F*_1_ = +7.16 points in favor of RF; adjusted *p* = 0.02). Positive Δ*F*_1_ values signify that the first-listed model performs better, while negative values signify that the second model performs better. All other *F*_1_ pairs, including RF vs. SVM (+0.27, adjusted *p* = 1.00), RF vs. XGBoost (+0.85, 1.00), and RF vs. MLP (+0.95, 1.00), were non-significant after multiple-comparison control, indicating that RF, SVM, and XGBoost are statistically indistinguishable on this endpoint. For recall (sensitivity), KNN had significantly lower values than MLP, RF, SVM, and XGBoost (Δ = −17.86, −17.14, −18.21, −19.29 points; adjusted *p* ≤ 0.004); Bagging exceeded KNN (+7.50; adjusted *p* = 0.035) but remained significantly below MLP, RF, SVM, and XGBoost (Δ = −10.36 to −11.79; adjusted *p* ≤ 0.041). For specificity, KNN exceeded MLP, RF, SVM, and XGBoost (+10.2, +8.0, +9.0, +11.4 points; adjusted *p* ≤ 0.005), and Bagging exceeded XGBoost (+6.40; adjusted *p* = 0.050); other pairs were non-significant after correction. For precision, KNN exceeded MLP, RF, SVM, and XGBoost (+12.49, +11.11, +12.10, +15.62; adjusted *p* ≤ 0.022); remaining contrasts were non-significant. For G-mean, RF exceeded Bagging (+4.55; adjusted *p* = 0.030), and KNN was lower than MLP, RF, SVM, and XGBoost (Δ = −6.20 to −7.19; adjusted *p* ≤ 0.035); other pairs did not reach significance. For MCC and accuracy, no pairwise differences survived Holm correction (minimum adjusted *p* ≈ 0.81). Detailed pairwise results for all metrics beyond *F*_1_ are consolidated in Supplementary Table S3.

**Table 3:** Pairwise Wilcoxon–Holm comparisons for *F*_1_ across 20 paired outer repetitions.

Pairwise Wilcoxon–Holm test results (20 repetitions)				
Comparison	Δ *F*_1_ (mean points)	Wilcoxon *p*	Holm-adj. *p*	Significance
Bagging vs. KNN	2.32	0.1712	1.00	ns
Bagging vs. MLP	−3.90	0.0641	0.58	ns
Bagging vs. RF	−4.85	0.0094	0.12	ns
Bagging vs. SVM	−4.58	0.0442	0.44	ns
Bagging vs. XGBoost	−4.00	0.0836	0.67	ns
KNN vs. MLP	−6.21	0.0136	0.15	ns
KNN vs. RF	−7.16	0.0014	0.02	*
KNN vs. SVM	−6.90	0.0073	0.10	ns
KNN vs. XGBoost	−6.32	0.0126	0.15	ns
MLP vs. RF	−0.95	0.5460	1.00	ns
MLP vs. SVM	−0.68	0.6435	1.00	ns
MLP vs. XGBoost	−0.11	0.8248	1.00	ns
RF vs. SVM	0.27	0.9826	1.00	ns
RF vs. XGBoost	0.85	0.4204	1.00	ns
SVM vs. XGBoost	0.58	0.6475	1.00	ns

ns = not significant (*p* ≥ 0.05); * = significant.

Feature selection stability analysis identified a consistent subset of morphometric variables with high recurrence across the 20 outer repetitions (Table 4). Four features, nerve transverse diameter (porus trigeminus), nerve vertical diameter (porus trigeminus), Meckel cave area (axial), and nerve vertical diameter (root) were selected in all repetitions by every model, indicating strong cross-architecture importance and supporting biological plausibility rather than model-specific artifacts. Additional features such as Meckel cave area (coronal), sagittal angle, and Meckel cave height also exhibited high selection rates (>85%) in most classifiers, reflecting their secondary relevance to model discrimination. Cisternal length and nerve transverse diameter (root) were selected in most runs, with rates varying by model (≈50–90% overall and up to ≈90% in XGBoost); angular measures (trigeminoclival and cisternopontine) showed greater between-model variability, while age was selected with moderate-to-high frequency across most models (≈45–70% of runs) and sex appeared only sporadically (<15%). The cross-model consistency of the top features suggests that the morphological narrowing of the trigeminal nerve at the porus trigeminus and the dimensional alterations of the Meckel cave are key determinants of classification performance. Detailed per-model selection distributions are illustrated in Supplementary Figure S1, which shows the top-15 selected features and their inclusion frequencies for each classifier.

**Table 4:** Frequency of feature selection across 20 outer repetitions for each model.

Feature	Bagging	KNN	MLP	Random Forest	SVM	XGBoost
Nerve transverse diameter (porus trigeminus)	20	20	20	20	20	20
Nerve vertical diameter (porus trigeminus)	20	20	20	20	20	20
Meckel cave area (axial)	20	20	20	20	20	20
Nerve vertical diameter (root)	20	20	20	20	20	20
Meckel cave area (coronal)	20	19	20	19	20	19
Sagittal angle	18	17	20	19	20	18
Meckel cave height	18	17	19	18	19	19
Cisternal length	13	14	14	15	15	18
Nerve transverse diameter (root)	12	11	10	14	14	18
Age	10	9	10	10	10	14
Trigeminoclival angle	10	6	8	10	9	8
Cisternopontine angle	8	4	6	8	6	10
Sex	1	3	0	3	1	2

Counts represent the number of times a feature was retained after SelectKBest filtering in each repetition.

Across the 20 outer repetitions, the best-trial hyperparameter configurations showed clear convergence trends within each model family.

In the case of Random Forest, a wide range of the number of trees (200–1,000) was tested, with the best configurations mainly around 300–700 trees. Feature sampling was usually done using sqrt or log2, and splitting criteria were mostly entropy or log loss, while Gini was almost never picked. The bootstrap option was alternately set true and false in different runs, and overall depth and splitting node thresholds were at moderate levels (max depth ≈ 8–30, min samples split ≈ 2–15, min samples leaf ≈ 1–9), which suggested that neither very shallow nor very deep trees were preferred.

For SVM, the radial-basis-function (RBF) kernel was in most cases the only kernel selected, followed by the linear kernel, and the polynomial kernels barely appeared at all. The regularization parameter C was a very large range of values (≈0.02–900), but the best run was at the middle to high end of the scale (≈2–50, sometimes even >100). The gamma parameter was most frequently set to auto or scale, with a small number of very small floating-point values, to prevent overfitting due to very high gamma values. Polynomials of degree around 4 were used to keep the kernel soft, and probabilistic outputs were always activated for reliable score estimation.

MLP had two major scenarios: (1) the LBFGS solver with hidden-layer layouts of (20, 10, 5), (20, 10), or (15, 15, 5) and (2) the Adam solver with similar layouts. ReLU was the standard activation, followed by tanh, and the logistic function was overhead on occasion only. The regularization coefficient, alpha, was set at the range of 1 × 10^−5^ to 1 × 10^−3^ most of the time, and the max iteration period was specified as 700 to 1,200. The learning rate was mostly adaptive with the batch size varying from 16 to 64 when Adam was applied.

For KNN, the number of neighbors ranged from 3 to 25, with the most optimal models selecting 6–14 neighbors. Distance-based weighting was favored over uniform weighting, and the metric was typically Minkowski (*p* = 1–2), with Manhattan and Euclidean metrics appearing in some cases. The leaf size ranged from 10 to 59, with mid-range values slightly more common.

For Bagging, two base learners were observed: decision trees and KNN. In tree-based variants, the number of estimators ranged from 30 to 160, with relatively high maximum samples and bootstrap features. In KNN-based variants, 3–10 neighbors and uniform or distance weights were most frequently used. The bootstrap and bootstrap features flags varied between runs, but bootstrap = true was slightly more common.

For XGBoost, the number of trees ranged from 200 to 1,000, with depths of 2–12 and learning rates between 1 × 10^−3^ and 3 × 10^−1^, most commonly converging to 0.05–0.2. The subsample and colsample_bytree ratios were typically 0.7–0.9, while min child weight ranged between 0.5 and 5. The gamma term was usually small (≈0–0.15), and both reg alpha and reg lambda remained near zero to moderate values. The scale pos weight parameter was tuned around the empirical imbalance ratio, within a 0.5–2× range of the negative-to-positive sample proportion.

Overall, these consistent hyperparameter patterns across models demonstrate that the Optuna-based search successfully identified stable regions of the parameter space, avoiding overly complex or underfitted configurations. A detailed mode and typical-range summary of all best-trial hyperparameters is provided in Supplementary Table S4, which lists the most frequent (mode) and interquartile-range (≈IQR) values observed across the 20 outer repetitions for each model family.

**Figure 6:**
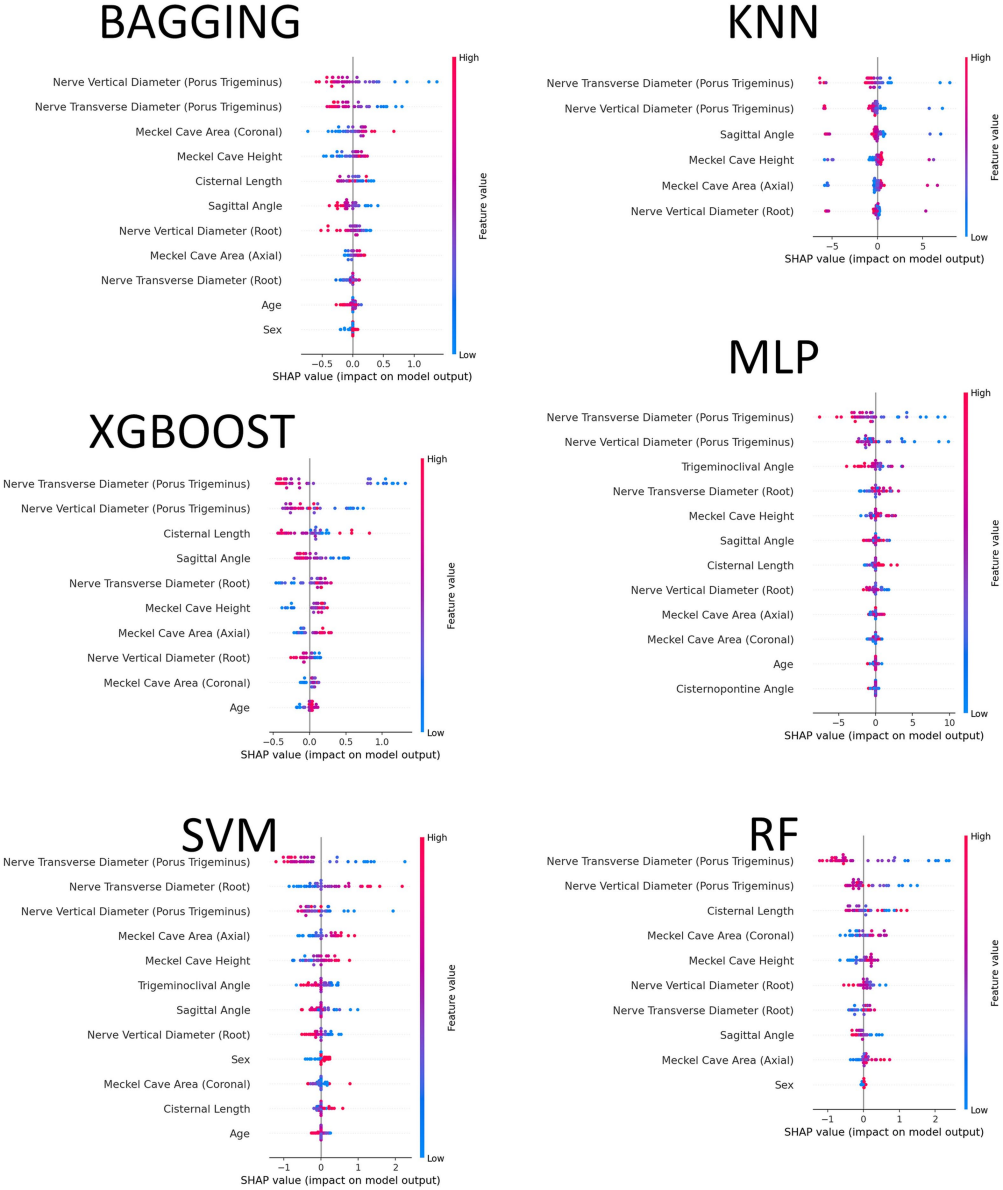
Global feature attributions from SHAP beeswarm plots for the six classifiers (Bagging, KNN, MLP, Random Forest, SVM, XGBoost), showing the distribution and direction of each feature’s contribution to the model output for the median run. Points represent individual test instances, colored by feature value (pink indicates high values, blue indicates low values).

Placing the median run (11th order statistic) side by side for all six models, the SHAP beeswarms in Figure 6 show a strikingly consistent global importance profile: measurements of the trigeminal nerve at the porus trigeminus (transverse and vertical diameters) dominate across models, followed by Meckel cave area (axial and coronal) and the root-level vertical diameter; second-tier contributors include Meckel cave height, sagittal angle, and cisternal length. The color-position patterns reveal a similar trend to those observed in univariate analysis: nerve diameters marked with lower values get predicted to belong to the symptomatic class more, while the ones with the higher values get predicted to belong to the control class more; the contrary effect is seen in Meckel cave measurements, as larger areas increase the likelihood of a symptomatic diagnosis. The nuances specific to models are not substantial: the use of tree ensemble methods (RF, XGBoost, Bagging) results in slightly tighter SHAP spreads for nerve diameter features, while SVM/MLP methods have wider spreads for angular and cave measurements, yet the essential feature ranking across models remains the same.

**Figure 7:**
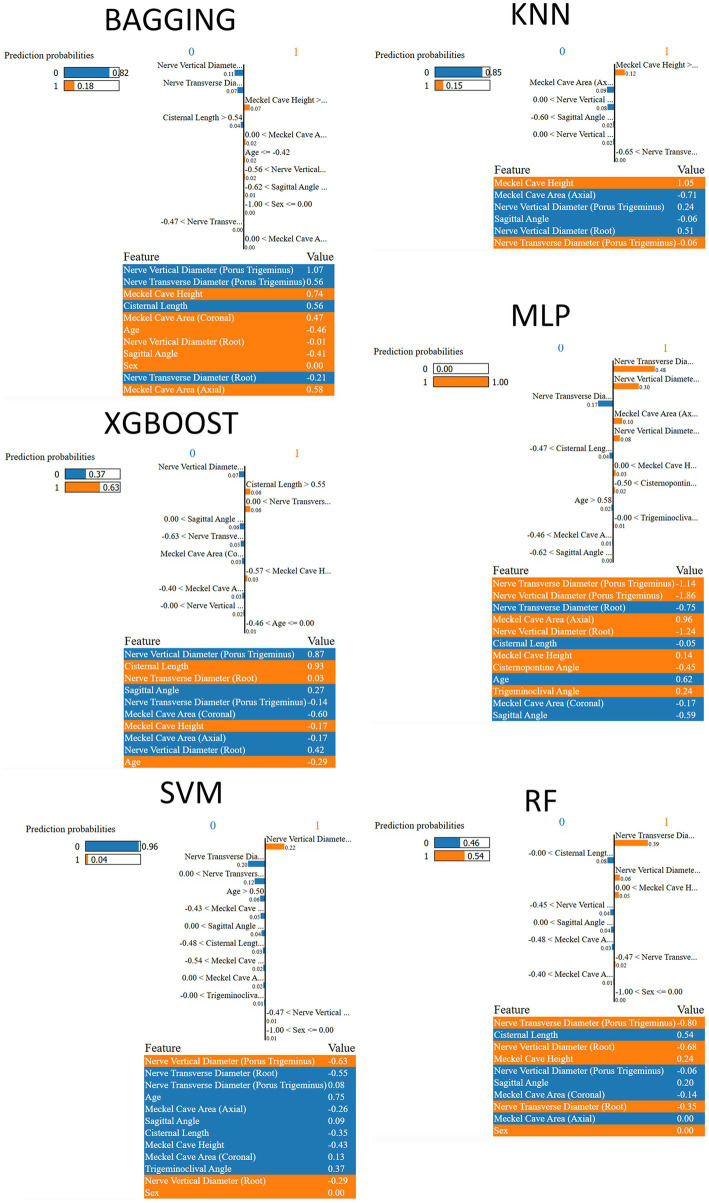
Local feature attributions from LIME explanations for the same test instance analyzed in the SHAP beeswarms (Figure 6), comparing local decision logic across the six models. Bars show the direction and magnitude of each feature’s contribution to the predicted probability.

Local, case-level explanations in Figure 7 (LIME) were computed using the same median-run test sample as in Figure 6, enabling a direct comparison between global and local levels. The top LIME attributions align with the SHAP profiles: the porus-level diameters and Meckel cave measurements account for most of the probability mass, with the sign of each contribution matching the SHAP direction (reduced nerve diameters increasing the predicted probability of the symptomatic class, and larger cave measurements exerting the opposite effect). This agreement across six distinct model families supports the notion that the learned decision logic is driven by the same small set of morphometric variables, rather than model-specific artifacts.

For robustness, Supplementary Figure S2 presents SHAP beeswarms for the five runs centered around the median (median ±2), demonstrating that the global importance pattern and directionality remain stable across nearby repetitions. Using the same test individual across those runs, Supplementary Figure S3 provides the corresponding LIME explanations, which reproduce the same set of leading contributors and signs observed in the main figures.

## 4 Discussion

This study supports a broader etiological framework for TN in which non-vascular morphometric variations of the prepontine cistern act as independent or synergistic risk factors alongside neurovascular compression. Across 20 outer repetitions and six model families, we observed a stable morphometric signature characterized by thinner trigeminal nerve diameters, larger Meckel cave areas and height, and smaller sagittal angles with shorter cisternal lengths. Such geometry is biologically plausible because it can lower the threshold for focal demyelination and ectopic excitability even when a compressive vessel is absent. The segmentation and measurement workflow demonstrated high repeatability and was standardized end-to-end, helping ensure that the observed differences reflected biology rather than measurement variability.

Our findings align with previous morphometric MRI studies, which report smaller trigeminal nerves and narrower prepontine spaces in patients with trigeminal neuralgia, although they differ in methodological rigor and reproducibility. Cheng et al. (12) and Ha et al. (13) described nerve atrophy and smaller trigeminal-pontine angles, consistent with our observation of thinner nerves and a smaller sagittal angle; cisternopontine and trigeminoclival angles were not significantly different after Holm correction in our cohort. Hardaway et al. (14) emphasized sex-related posterior fossa differences; however, sex appeared infrequently in our feature selection and XAI analyses, suggesting a minor contribution in our cohort. Kundakçı et al. (15) confirmed morphometric variation of the trigeminal nerve and adjacent structures, and Branstetter et al. (16) highlighted the discriminative value of the sagittal angle at the porus trigeminus, which showed intermediate importance in our results. Prior reports have largely relied on manual or semi-quantitative measurements, which are susceptible to observer variability. In contrast, standardized, AI-assisted morphometry and a leakage-free nested pipeline improved reproducibility and objectivity, providing a statistically controlled validation of qualitative trends reported earlier (12–16), and remaining compatible with observations that TN can manifest without demonstrable NVC (6).

Interpretability and stability analyses converged on a compact, physiologically coherent feature set. SHAP and LIME consistently prioritized porus-level nerve diameters and Meckel’s cave measures, which were also the variables with the largest pre-classification group shifts, indicating that the learned signal is not a classifier-specific artifact. Selection-frequency analyses across outer repetitions supported this convergence and suggested that the morphometric signature is reproducible across resamples and models.

From a model-selection perspective, families captured essentially the same signal but with complementary operating characteristics. Support vector machines yielded threshold-independent summaries with the highest PR-AUC and a competitive ROC-AUC, supporting reliable ranking for screening. Random forests balance threshold-dependent metrics, including *F*_1_, accuracy, G-mean, and MCC, making them attractive when decisions are anchored to a conventional probability cut-point. MLP and XGBoost offered the highest recalls when minimizing false negatives is a priority, whereas KNN and Bagging traded recall for higher specificity and precision, a profile more consistent with confirmatory use. Inferentially, the Friedman test on *F*_1_ detected a global difference, but only the random-forest versus KNN contrast remained significant after Holm correction; pairwise differences among random forest, SVM, and XGBoost were not significant, consistent with their overlapping PR and ROC bands. These results suggest a pragmatic deployment strategy that selects both the algorithm and operating threshold to match the clinical use case, followed by calibration and monitoring using prospective data. The complete set of pairwise Wilcoxon–Holm results and global tests across metrics is summarized in Supplementary Table S3.

Clinically, an exclusive focus on vascular contact risks misclassification and suboptimal treatment allocation. Integrating non-vascular morphometry, especially porus level diameters and Meckel cave size, into diagnostic pathways could refine selection for microvascular decompression versus alternative interventions and may help anticipate recurrence in anatomically susceptible patients. In parallel, model choice should be guided by clinical priorities: SVM for robust ranking in screening workflows, random forests for balanced performance at a fixed threshold, MLP or XGBoost when reducing false negatives is paramount, and KNN or Bagging when minimizing false positives is critical and conservative operating points are acceptable.

Clinically, an exclusive focus on vascular contact risks misclassification and suboptimal treatment allocation. In our XAI analyses, the morphometrics most consistently prioritized by SHAP/LIME particularly porus-level trigeminal nerve diameters and Meckel’s cave size measures are clinically “actionable” because they are directly measurable on routine high-resolution posterior fossa MRI and can be reported in a standardized manner. These quantitative descriptors may be especially useful in equivocal scenarios, such as Grade 1 contact where clinical significance is uncertain, by providing objective morphometric context to support diagnostic stratification alongside the clinical phenotype. Integrating non-vascular morphometry, especially porus level diameters and Meckel cave size, into diagnostic pathways could refine selection for microvascular decompression versus alternative interventions and may help anticipate recurrence in anatomically susceptible patients. Importantly, these measurements are intended to complement rather than replace established diagnostic criteria and surgical indications. In parallel, model choice should be guided by clinical priorities: SVM for robust ranking in screening workflows, random forests for balanced performance at a fixed threshold, MLP or XGBoost when reducing false negatives is paramount, and KNN or Bagging when minimizing false positives is critical and conservative operating points are acceptable.

This work has limitations. The single-center, retrospective design may limit generalizability across scanners, sequences, and patient mix; therefore, multicenter external validation is needed to confirm transportability and performance stability. The cross-sectional nature precludes causal inference about whether morphometric differences precede symptom onset. Although the nested workflow mitigated class imbalance, larger prospective cohorts are needed to rule out residual effects. Only structural MRI was analyzed. Incorporating diffusion tensor imaging, tractography, and network-level assessments may reveal microstructural and functional correlates that clarify the vulnerability of the trigeminal system.

Extending the pipeline to diffusion-based microstructural and tractography-based features may clarify mechanisms of nerve vulnerability and improve prognostication. Ultimately, prospective trials evaluating whether morphometry-aware decision support enhances referral and treatment outcomes will be crucial to realizing the clinical value of these findings.

## 5 Conclusion

This study demonstrates that non-vascular prepontine cistern anatomy significantly contributes to the pathophysiology of iTN. Using a standardized, machine-learning-based morphometric workflow, we identified a reproducible anatomical signature characterized by thinner trigeminal nerve diameters (especially at the porus), smaller sagittal angles, larger Meckel’s cave areas, and shorter cisternal lengths, which distinguishes iTN from controls. Across 20 paired outer repetitions, multiple classifier families captured this signal with comparable discrimination, while offering complementary operating characteristics. Integrating vascular and non-vascular morphometry into diagnostic pathways may refine patient stratification, support threshold-aware model deployment, and inform individualized management.

## Data Availability

The raw data supporting the conclusions of this article will be made available by the authors, without undue reservation.
