# Supplementary material for: A comprehensive evaluation of non-vascular prepontine cistern anatomy influencing trigeminal nerve vulnerability using machine learning-based morphometric analysis
Source: Front Med (Lausanne). 2026 Mar 3;13:1745815. doi: 10.3389/fmed.2026.1745815 (PMC12992038; doi:10.3389/fmed.2026.1745815)

**Supplementary Table S1.** Complete results of feature-level statistical comparisons between the Asymptomatic Control (class 0) and Symptomatic iTN (class 1) groups.
The table reports detailed outcomes for each numerical and categorical variable, including sample sizes, class-specific means and medians, mean difference (Δ Mean = Class 1 − Class 0), direction of change, two-tailed *t*-test and Mann–Whitney U (*MWU*) *p*-values, variance homogeneity (*Levene’s p*), effect sizes (Cohen’s *d*, Cliff’s δ, φ), and multiple-comparison–adjusted *p*-values (FDR–Benjamini–Hochberg and Holm). Significance flags (*sig p < 0.01* and *sig FDR < 0.01*) indicate which features remained statistically different after correction. Negative Δ Mean and effect-size values denote lower central tendencies in the symptomatic (CLASS 1) group.

| **feature** | Nerve Transverse Diameter (Porus Trigeminus) | Nerve Vertical Diameter (Porus Trigeminus) | Meckel Cave Area (Axial) | Nerve Vertical Diameter (Root) | Meckel Cave Area (Coronal) | Sagittal Angle | Meckel Cave Height | Cisternal Length | Trigeminoclival Angle | Age | Nerve Transverse Diameter (Root) | Cisternopontine Angle | Sex |
| --- | --- | --- | --- | --- | --- | --- | --- | --- | --- | --- | --- | --- | --- |
| **type** | continuous | continuous | continuous | continuous | continuous | continuous | continuous | continuous | continuous | continuous | continuous | continuous | categorical(2x2) |
| **n_class1** | 71 | 71 | 71 | 71 | 71 | 71 | 71 | 71 | 71 | 71 | 71 | 71 | 71 |
| **n_class0** | 120 | 120 | 120 | 120 | 120 | 120 | 120 | 120 | 120 | 120 | 120 | 120 | 120 |
| **mean_class1** | 2.65 | 1.77 | 0.70 | 1.75 | 0.57 | 131.85 | 2.44 | 6.42 | 49.21 | 56.13 | 2.18 | 39.72 |  |
| **mean_class0** | 3.62 | 2.18 | 0.55 | 2.06 | 0.47 | 137.18 | 2.15 | 7.05 | 51.42 | 51.75 | 2.33 | 41.29 |  |
| **median_class1** | 2.55 | 1.72 | 0.64 | 1.72 | 0.53 | 132.90 | 2.39 | 6.25 | 50.10 | 57.00 | 2.18 | 40.10 |  |
| **median_class0** | 3.59 | 2.11 | 0.53 | 2.05 | 0.45 | 138.30 | 2.18 | 7.04 | 51.85 | 52.00 | 2.25 | 41.10 |  |
| **delta_mean_1_0** | -0.96 | -0.41 | 0.15 | -0.32 | 0.11 | -5.32 | 0.28 | -0.63 | -2.20 | 4.38 | -0.15 | -1.57 |  |
| **direction** | class1<class0 | class1<class0 | class1>class0 | class1<class0 | class1>class0 | class1<class0 | class1>class0 | class1<class0 | class1<class0 | class1>class0 | class1<class0 | class1<class0 | class1<class0 |
| **t_p_two_sided** | 3.04799E-13 | 1.03795E-07 | 5.59556E-06 | 3.5615E-06 | 0.000303519 | 0.001005971 | 0.001371908 | 0.016076562 | 0.130048808 | 0.055511774 | 0.073250796 | 0.271671273 |  |
| **mw_p_two_sided** | 1.82398E-14 | 2.12952E-10 | 2.19655E-06 | 1.27868E-05 | 0.000102195 | 0.001147675 | 0.002248713 | 0.006515059 | 0.148836668 | 0.156833805 | 0.160604261 | 0.284681412 |  |
| **mw_p_one_sided_1_lt_0** | 9.11991E-15 | 1.06476E-10 | 0.999998916 | 6.3934E-06 | 0.999949469 | 0.000573837 | 0.998885756 | 0.003257529 | 0.074418334 | 0.921979123 | 0.08030213 | 0.142340706 |  |
| **levene_p** | <1E−3 | 0.02 | 0.01 | 0.83 | 0.01 | 0.01 | 0.37 | <1E−3 | 0.64 | 0.03 | 0.82 | 0.40 |  |
| **cohen_d** | -1.31 | -0.89 | 0.75 | -0.72 | 0.59 | -0.52 | 0.49 | -0.38 | -0.23 | 0.28 | -0.27 | -0.17 |  |
| **cliffs_delta** | -0.66 | -0.55 | 0.41 | -0.38 | 0.34 | -0.28 | 0.26 | -0.24 | -0.13 | 0.12 | -0.12 | -0.09 |  |
| **fisher_odds_ratio** |  |  |  |  |  |  |  |  |  |  |  |  | 1.22 |
| **fisher_p_two_sided** |  |  |  |  |  |  |  |  |  |  |  |  | 0.64 |
| **phi_effect** |  |  |  |  |  |  |  |  |  |  |  |  | 0.05 |
| **p_primary** | 1.82398E-14 | 2.12952E-10 | 2.19655E-06 | 1.27868E-05 | 0.000102195 | 0.001147675 | 0.002248713 | 0.006515059 | 0.148836668 | 0.156833805 | 0.160604261 | 0.284681412 | 0.641323164 |
| **p_FDR_BH** | 2.37118E-13 | 1.38419E-09 | 9.51838E-06 | 4.15571E-05 | 0.000265708 | 0.002486629 | 0.004176181 | 0.01058697 | 0.189805036 | 0.189805036 | 0.189805036 | 0.308404863 | 0.641323164 |
| **p_Holm** | 2.37118E-13 | 2.55542E-09 | 2.4162E-05 | 0.000127868 | 0.000919758 | 0.009181398 | 0.01574099 | 0.039090352 | 0.74418334 | 0.74418334 | 0.74418334 | 0.74418334 | 0.74418334 |
| **sig_p_lt_0_01** | TRUE | TRUE | TRUE | TRUE | TRUE | TRUE | TRUE | TRUE | FALSE | FALSE | FALSE | FALSE | FALSE |
| **sig_FDR_lt_0_01** | TRUE | TRUE | TRUE | TRUE | TRUE | TRUE | TRUE | FALSE | FALSE | FALSE | FALSE | FALSE | FALSE |

**Supplementary Table S2**. Hyperparameter search spaces and common settings used in the nested Optuna workflow.

| **OVERVIEW** | | | | |
| --- | --- | --- | --- | --- |
| **Parameter** | **Type** | **Search Space (Bounds / Choices)** | **Step / Prior** | **Notes** |
| Optimizer | Optuna Sampler | Tree-structured Parzen Estimator (TPE) |  | Bayesian sampler |
| Pruner | Early stopping | Median Pruner | warm-up=10 trials | Prunes below running median |
| Trials per model | Integer | 150 |  | Default |
| Splits | StratifiedShuffleSplit | Outer test=20%; Inner train≈65%, val≈15% |  | Same splits reused across models |
| Positive class | Label | CLASS=1 (class1) |  | Metrics computed w.r.t. CLASS=1 |
| Feature selector | SelectKBest | k ∈ [5, 12] | Integer | ANOVA F-statistic; after preprocessing |
|  |  |  |  |  |
| **Random Forest** | | | | |
| **Parameter** | **Type** | **Search Space (Bounds / Choices)** | **Step / Prior** | **Notes** |
| n_estimators | Integer | [200, 1000] | step=100 |  |
| max_depth | Integer | [2, 30] |  |  |
| min_samples_split | Integer | [2, 20] |  |  |
| min_samples_leaf | Integer | [1, 10] |  |  |
| max_features | Categorical | ['sqrt','log2',None] |  |  |
| criterion | Categorical | ['gini','entropy','log_loss'] |  |  |
| bootstrap | Categorical | [True, False] |  |  |
| class_weight | Fixed | balanced |  | Inverse-frequency weighting |
| n_jobs | Fixed | -1 |  | Parallel |
| random_state | Seed | rng_seed |  | Per repetition |
|  |  |  |  |  |
| **SVM** | | | | |
| **Parameter** | **Type** | **Search Space (Bounds / Choices)** | **Step / Prior** | **Notes** |
| kernel | Categorical | ['linear','rbf','poly'] |  |  |
| C | Float | [1e-3, 1e3] | log-uniform |  |
| gamma_mode | Categorical | ['scale','auto','float'] |  | If 'float', sample gamma |
| gamma | Float (if gamma_mode='float') | [1e-4, 1.0] | log-uniform |  |
| degree | Integer (if kernel='poly') | [2, 5] |  |  |
| class_weight | Fixed | balanced |  |  |
| probability | Fixed | True |  | Enable probabilistic outputs |
| random_state | Seed | rng_seed |  |  |
|  |  |  |  |  |
| **MLP** | | | | |
| **Parameter** | **Type** | **Search Space (Bounds / Choices)** | **Step / Prior** | **Notes** |
| hidden_layer_sizes | Categorical | [(20,10,5),(15,15,5),(20,15,5),(20,10)] |  |  |
| activation | Categorical | ['relu','tanh','logistic'] |  |  |
| alpha | Float | [1e-5, 1e-1] | log-uniform | L2 penalty |
| solver | Categorical | ['adam','lbfgs'] |  |  |
| max_iter | Integer | [400, 1200] | step=100 |  |
| learning_rate | Categorical (if solver='adam') | ['constant','adaptive'] |  |  |
| learning_rate_init | Float (if solver='adam') | [1e-5, 1e-3] | log-uniform |  |
| beta_1 | Float (if solver='adam') | [0.7, 0.99] | uniform |  |
| beta_2 | Float (if solver='adam') | [0.9, 0.999] | uniform |  |
| early_stopping | Categorical (if solver='adam') | [True, False] |  |  |
| batch_size | Categorical (if solver='adam') | [16, 32, 64] |  |  |
| random_state | Seed | rng_seed |  |  |
|  |  |  |  |  |
| **XGBoost** | | | | |
| **Parameter** | **Type** | **Search Space (Bounds / Choices)** | **Step / Prior** | **Notes** |
| n_estimators | Integer | [200, 1000] | step=100 |  |
| max_depth | Integer | [2, 10] |  |  |
| learning_rate | Float | [1e-3, 3e-1] | log-uniform |  |
| subsample | Float | [0.5, 1.0] | uniform |  |
| colsample_bytree | Float | [0.5, 1.0] | uniform |  |
| min_child_weight | Float | [1e-1, 10.0] | log-uniform |  |
| gamma | Float | [0.0, 5.0] | uniform |  |
| reg_alpha | Float | [1e-8, 1.0] | log-uniform |  |
| reg_lambda | Float | [1e-3, 10.0] | log-uniform |  |
| scale_pos_weight | Float | [0.5×spw_base, 2.0×spw_base] | log-uniform | spw_base = neg/pos ratio |
| tree_method | Fixed | hist |  | Histogram-based grower |
| eval_metric | Fixed | logloss |  |  |
| n_jobs | Fixed | -1 |  | Parallel |
| random_state | Seed | rng_seed |  |  |
|  |  |  |  |  |
| **KNN** | | | | |
| **Parameter** | **Type** | **Search Space (Bounds / Choices)** | **Step / Prior** | **Notes** |
| n_neighbors | Integer | [1, 30] |  |  |
| weights | Categorical | ['uniform','distance'] |  |  |
| p | Integer | [1, 2] |  | Minkowski order |
| leaf_size | Integer | [10, 60] |  |  |
| metric | Categorical | ['minkowski','euclidean','manhattan'] |  |  |
|  |  |  |  |  |
| **BAGGING** | | | | |
| **Parameter** | **Type** | **Search Space (Bounds / Choices)** | **Step / Prior** | **Notes** |
| base_estimator | Categorical | ['tree','knn'] |  |  |
| n_estimators | Integer | [10, 200] | step=10 |  |
| max_samples | Float | [0.5, 1.0] | uniform |  |
| max_features | Float | [0.5, 1.0] | uniform |  |
| bootstrap | Categorical | [True, False] |  |  |
| bootstrap_features | Categorical | [False, True] |  |  |
| n_jobs | Fixed | -1 |  | Parallel |
| random_state | Seed | rng_seed |  |  |
|  |  |  |  |  |
| **BAGGING-TreeBase** | | | | |
| **Parameter** | **Type** | **Search Space (Bounds / Choices)** | **Step / Prior** | **Notes** |
| max_depth | Integer | [1, 15] |  |  |
| min_samples_split | Integer | [2, 10] |  |  |
| min_samples_leaf | Integer | [1, 5] |  |  |
| class_weight | Fixed | balanced |  |  |
|  |  |  |  |  |
| **BAGGING-KNNBase** | | | | |
| **Parameter** | **Type** | **Search Space (Bounds / Choices)** | **Step / Prior** | **Notes** |
| n_neighbors | Integer | [3, 15] |  |  |
| weights | Categorical | ['uniform','distance'] |  |  |
|  |  |  |  |  |
| **Feature Selection** | | | | |
| **Parameter** | **Type** | **Search Space (Bounds / Choices)** | **Step / Prior** | **Notes** |
| k_best | Integer | [5, 12] |  | SelectKBest (ANOVA F-statistic) |

**Supplementary Table S3.** Complete pairwise Wilcoxon–Holm results across 20 paired outer repetitions for all performance metrics (Accuracy, G-mean, MCC, Precision, Recall/Sensitivity, and Specificity).

| **Metric** | **χ² (Friedman statistic)** | **df** | **p-value** | **k (models)** | **n (repetitions)** | **Significance** |
| --- | --- | --- | --- | --- | --- | --- |
| Specificity | 35.27 | 5 | 1.33 × 10⁻⁶ | 6 | 20 | *** |
| Precision | 25.94 | 5 | 9.15 × 10⁻⁵ | 6 | 20 | *** |
| Recall | 49.17 | 5 | 2.05 × 10⁻⁹ | 6 | 20 | *** |
| F1 | 16.47 | 5 | 0.0056 | 6 | 20 | ***** |
| MCC | 3.4 | 5 | 0.639 | 6 | 20 | ns |
| G-mean | 23.49 | 5 | 0.00027 | 6 | 20 | ***** |
| Accuracy | 1.48 | 5 | 0.915 | 6 | 20 | ns |
| ns = not significant (p ≥ 0.05); * p < 0.05; *** p < 0.001. | | | | | | |

| **Pairwise Wilcoxon–Holm test results (20 repetitions)** | | | | |
| --- | --- | --- | --- | --- |
| **Comparison** | **Δ Accuracy (mean points)** | **Wilcoxon p** | **Holm-adj p** | **Significance** |
| Bagging vs. KNN | -0.51 | 1.0000 | 1 | ns |
| Bagging vs. MLP | -0.38 | 0.8300 | 1 | ns |
| Bagging vs. RF | -1.54 | 0.2421 | 1 | ns |
| Bagging vs. SVM | -1.28 | 0.4550 | 1 | ns |
| Bagging vs. XGBoost | -0.13 | 0.8612 | 1 | ns |
| KNN vs. MLP | 0.13 | 0.9430 | 1 | ns |
| KNN vs. RF | -1.03 | 0.2243 | 1 | ns |
| KNN vs. SVM | -0.77 | 0.4233 | 1 | ns |
| KNN vs. XGBoost | 0.38 | 0.9546 | 1 | ns |
| MLP vs. RF | -1.15 | 0.3908 | 1 | ns |
| MLP vs. SVM | -0.90 | 0.5822 | 1 | ns |
| MLP vs. XGBoost | 0.26 | 0.9303 | 1 | ns |
| RF vs. SVM | 0.26 | 0.7234 | 1 | ns |
| RF vs. XGBoost | 1.41 | 0.0543 | 0.814 | ns |
| SVM vs. XGBoost | 1.15 | 0.2208 | 1 | ns |
|  |  |  |  |  |
| **Pairwise Wilcoxon–Holm test results (20 repetitions)** | | | | |
| **Comparison** | **ΔGmean (mean points)** | **Wilcoxon p** | **Holm-adj p** | **Significance** |
| Bagging vs. KNN | 2.64 | 0.0641 | 0.449 | ns |
| Bagging vs. MLP | -3.56 | 0.0298 | 0.268 | ns |
| Bagging vs. RF | -4.55 | 0.0023 | 0.030 | * |
| Bagging vs. SVM | -4.53 | 0.0215 | 0.215 | ns |
| Bagging vs. XGBoost | -4.15 | 0.0364 | 0.291 | ns |
| KNN vs. MLP | -6.20 | 0.0032 | 0.035 | * |
| KNN vs. RF | -7.19 | 0.0001 | 0.002 | * |
| KNN vs. SVM | -7.16 | 0.0019 | 0.027 | * |
| KNN vs. XGBoost | -6.79 | 0.0029 | 0.035 | * |
| MLP vs. RF | -0.99 | 0.4939 | 1 | ns |
| MLP vs. SVM | -0.96 | 0.4688 | 1 | ns |
| MLP vs. XGBoost | -0.59 | 0.9199 | 1 | ns |
| RF vs. SVM | 0.02 | 0.7771 | 1 | ns |
| RF vs. XGBoost | 0.40 | 0.7112 | 1 | ns |
| SVM vs. XGBoost | 0.37 | 0.7439 | 1 | ns |
| ns = not significant (p ≥ 0.05); * = significant. | | | | |
|  |  |  |  |  |
|  |  |  |  |  |
| **Pairwise Wilcoxon–Holm test results (20 repetitions)** | | | | |
| **Comparison** | **ΔMCC (mean points)** | **Wilcoxon p** | **Holm-adj p** | **Significance** |
| Bagging vs. KNN | -0.98 | 0.7475 | 1 | ns |
| Bagging vs. MLP | -2.20 | 0.3144 | 1 | ns |
| Bagging vs. RF | -3.62 | 0.2162 | 1 | ns |
| Bagging vs. SVM | -3.35 | 0.3300 | 1 | ns |
| Bagging vs. XGBoost | -1.13 | 0.6292 | 1 | ns |
| KNN vs. MLP | -1.23 | 0.4524 | 1 | ns |
| KNN vs. RF | -2.65 | 0.3884 | 1 | ns |
| KNN vs. SVM | -2.37 | 0.2774 | 1 | ns |
| KNN vs. XGBoost | -0.15 | 0.9359 | 1 | ns |
| MLP vs. RF | -1.42 | 0.5197 | 1 | ns |
| MLP vs. SVM | -1.14 | 0.7782 | 1 | ns |
| MLP vs. XGBoost | 1.07 | 0.5869 | 1 | ns |
| RF vs. SVM | 0.28 | 0.6474 | 1 | ns |
| RF vs. XGBoost | 2.50 | 0.1841 | 1 | ns |
| SVM vs. XGBoost | 2.22 | 0.2860 | 1 | ns |
| ns = not significant (p ≥ 0.05); * = significant. | | | | |
|  |  |  |  |  |
| **Pairwise Wilcoxon–Holm test results (20 repetitions)** | | | | |
| **Comparison** | **ΔPrecision (mean points)** | **Wilcoxon p** | **Holm-adj p** | **Significance** |
| Bagging vs. KNN | -7.48 | 0.0340 | 0.340 | ns |
| Bagging vs. MLP | 5.02 | 0.0642 | 0.577 | ns |
| Bagging vs. RF | 3.63 | 0.2514 | 1 | ns |
| Bagging vs. SVM | 4.63 | 0.1262 | 0.883 | ns |
| Bagging vs. XGBoost | 8.15 | 0.0249 | 0.274 | ns |
| KNN vs. MLP | 12.49 | 0.0018 | 0.022 | * |
| KNN vs. RF | 11.11 | 0.0011 | 0.014 | * |
| KNN vs. SVM | 12.10 | 0.0008 | 0.012 | * |
| KNN vs. XGBoost | 15.62 | 0.0003 | 0.005 | * |
| MLP vs. RF | -1.38 | 0.5731 | 1 | ns |
| MLP vs. SVM | -0.39 | 1.0000 | 1 | ns |
| MLP vs. XGBoost | 3.13 | 0.2121 | 1 | ns |
| RF vs. SVM | 1.00 | 0.4996 | 1 | ns |
| RF vs. XGBoost | 4.52 | 0.0778 | 0.622 | ns |
| SVM vs. XGBoost | 3.52 | 0.3061 | 1 | ns |
| ns = not significant (p ≥ 0.05); * = significant. | | | | |
|  |  |  |  |  |
| **Pairwise Wilcoxon–Holm test results (20 repetitions)** | | | | |
| **Comparison** | **Δ Recall (mean points)** | **Wilcoxon p** | **Holm-adj p** | **Significance** |
| Bagging vs. KNN | 7.50 | 0.0039 | 0.035 | * |
| Bagging vs. MLP | -10.36 | 0.0044 | 0.035 | * |
| Bagging vs. RF | -9.64 | 0.0023 | 0.023 | * |
| Bagging vs. SVM | -10.71 | 0.0059 | 0.041 | * |
| Bagging vs. XGBoost | -11.79 | 0.0019 | 0.021 | * |
| KNN vs. MLP | -17.86 | 0.0003 | 0.004 | * |
| KNN vs. RF | -17.14 | 0.0001 | 0.002 | * |
| KNN vs. SVM | -18.21 | 0.0003 | 0.004 | * |
| KNN vs. XGBoost | -19.29 | 0.0003 | 0.004 | * |
| MLP vs. RF | 0.71 | 0.7501 | 1 | ns |
| MLP vs. SVM | -0.36 | 0.7229 | 1 | ns |
| MLP vs. XGBoost | -1.43 | 0.5921 | 1 | ns |
| RF vs. SVM | -1.07 | 0.5866 | 1 | ns |
| RF vs. XGBoost | -2.14 | 0.1106 | 0.664 | ns |
| SVM vs. XGBoost | -1.07 | 0.8747 | 1 | ns |
| ns = not significant (p ≥ 0.05); * = significant. | | | | |
|  |  |  |  |  |
| **Pairwise Wilcoxon–Holm test results (20 repetitions)** | | | | |
| **Comparison** | **ΔSpecificity (mean points)** | **Wilcoxon p** | **Holm-adj p** | **Significance** |
| Bagging vs. KNN | -5.00 | 0.0104 | 0.097 | ns |
| Bagging vs. MLP | 5.20 | 0.0097 | 0.097 | ns |
| Bagging vs. RF | 3.00 | 0.1742 | 1 | ns |
| Bagging vs. SVM | 4.00 | 0.0450 | 0.315 | ns |
| Bagging vs. XGBoost | 6.40 | 0.0045 | 0.050 | * |
| KNN vs. MLP | 10.20 | 0.0004 | 0.005 | * |
| KNN vs. RF | 8.00 | 0.0004 | 0.005 | * |
| KNN vs. SVM | 9.00 | 0.0002 | 0.003 | * |
| KNN vs. XGBoost | 11.40 | 0.0003 | 0.004 | * |
| MLP vs. RF | -2.20 | 0.3408 | 1 | ns |
| MLP vs. SVM | -1.20 | 0.6568 | 1 | ns |
| MLP vs. XGBoost | 1.20 | 0.4048 | 1 | ns |
| RF vs. SVM | 1.00 | 0.4504 | 1 | ns |
| RF vs. XGBoost | 3.40 | 0.0370 | 0.296 | ns |
| SVM vs. XGBoost | 2.40 | 0.3177 | 1 | ns |
| ns = not significant (p ≥ 0.05); * = significant. | | | | |

**Supplementary Figure S1.** Top-15 selected features for each model (Bagging, KNN, MLP, Random Forest, SVM, XGBoost) with selection rates computed over 20 outer repetitions. Bars indicate the proportion of runs in which each feature was retained within the final model pipeline.
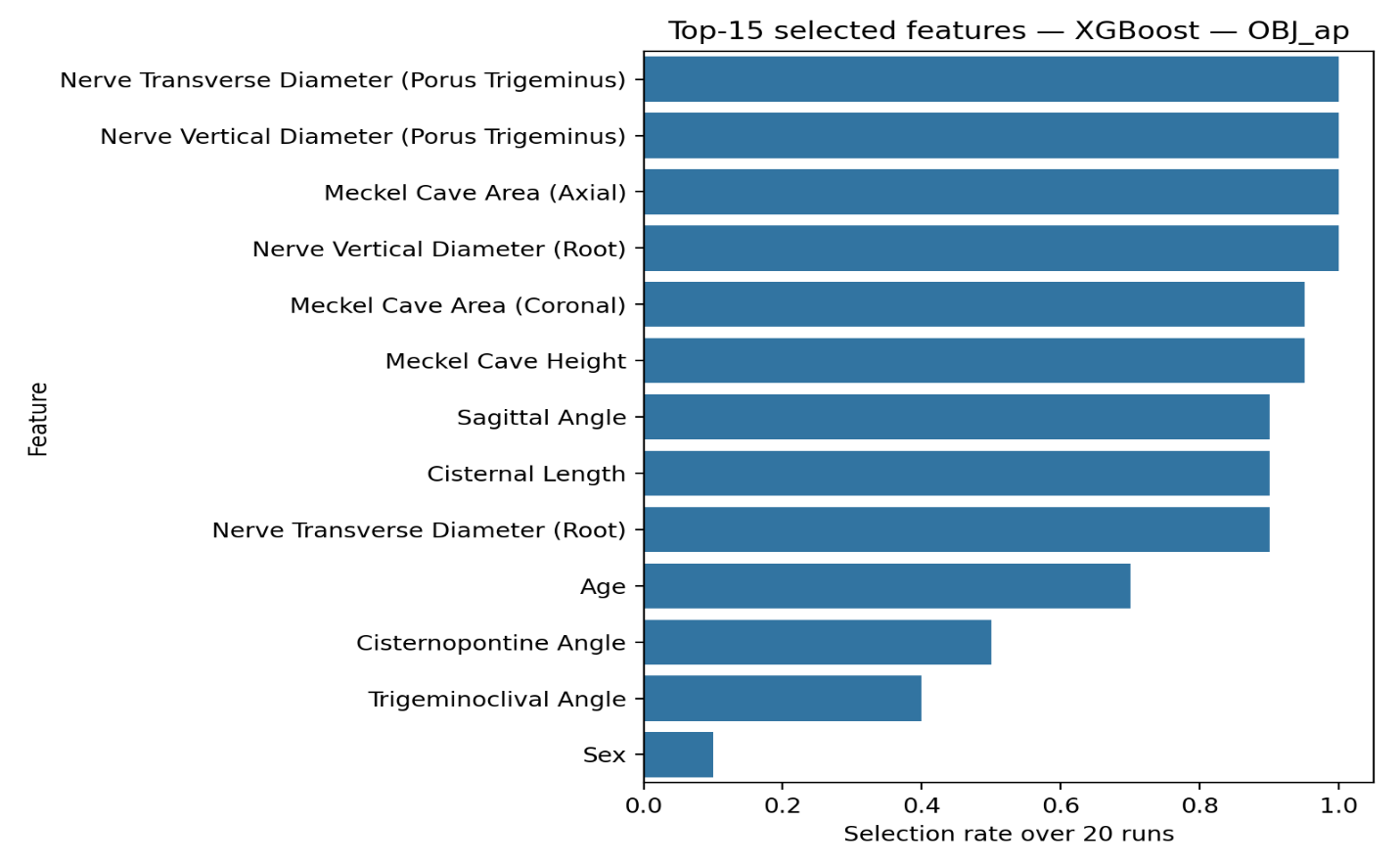

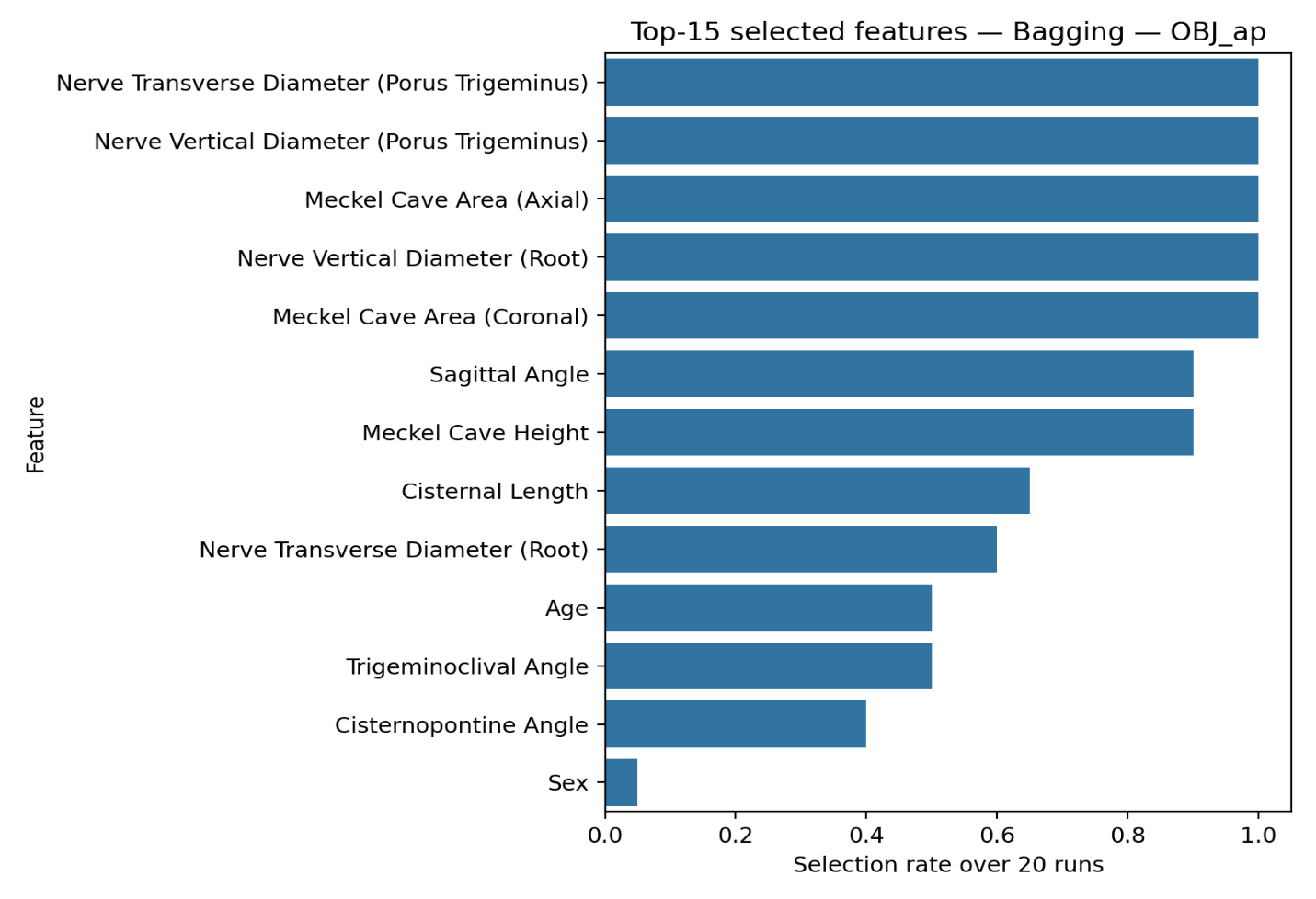

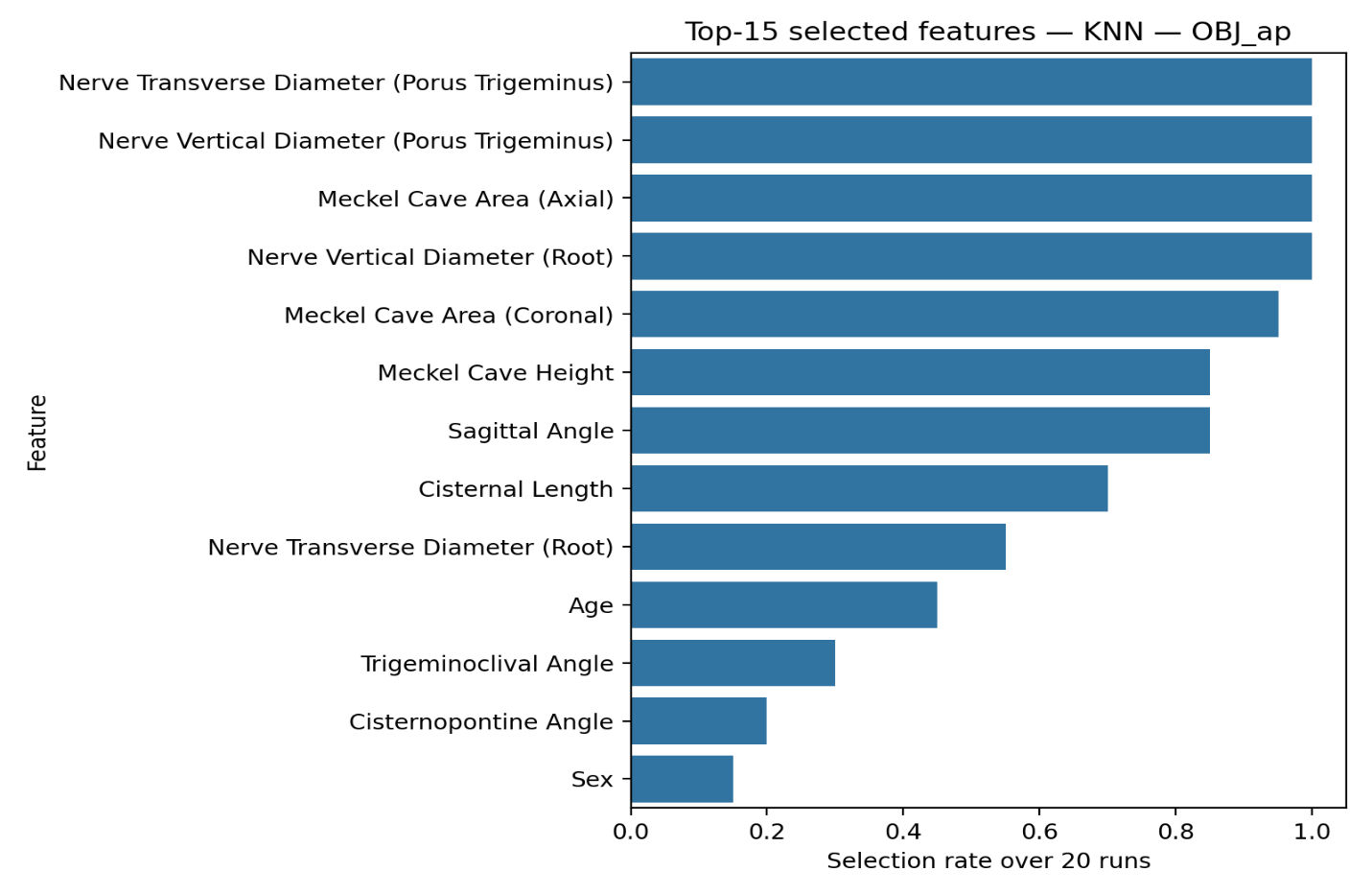

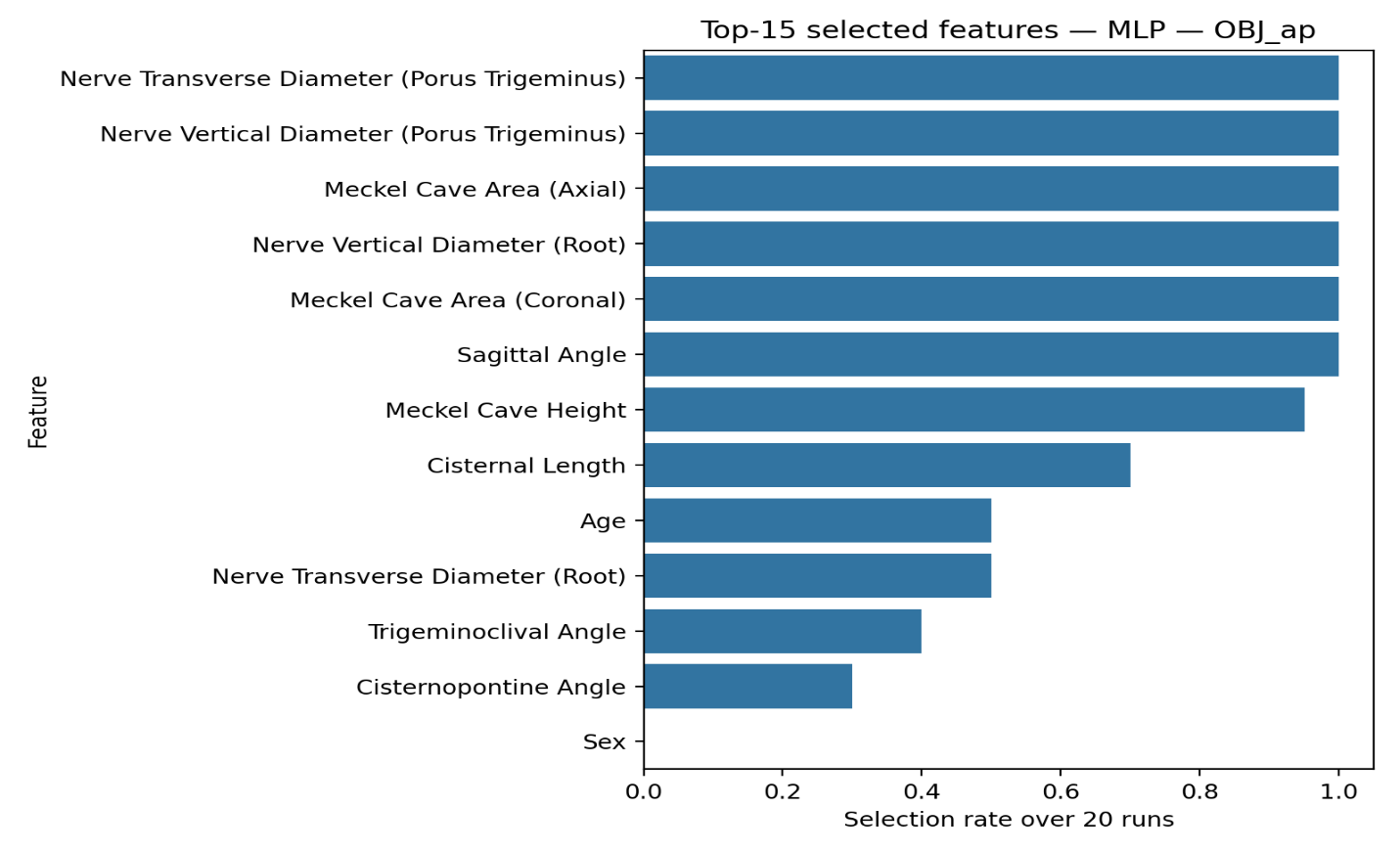

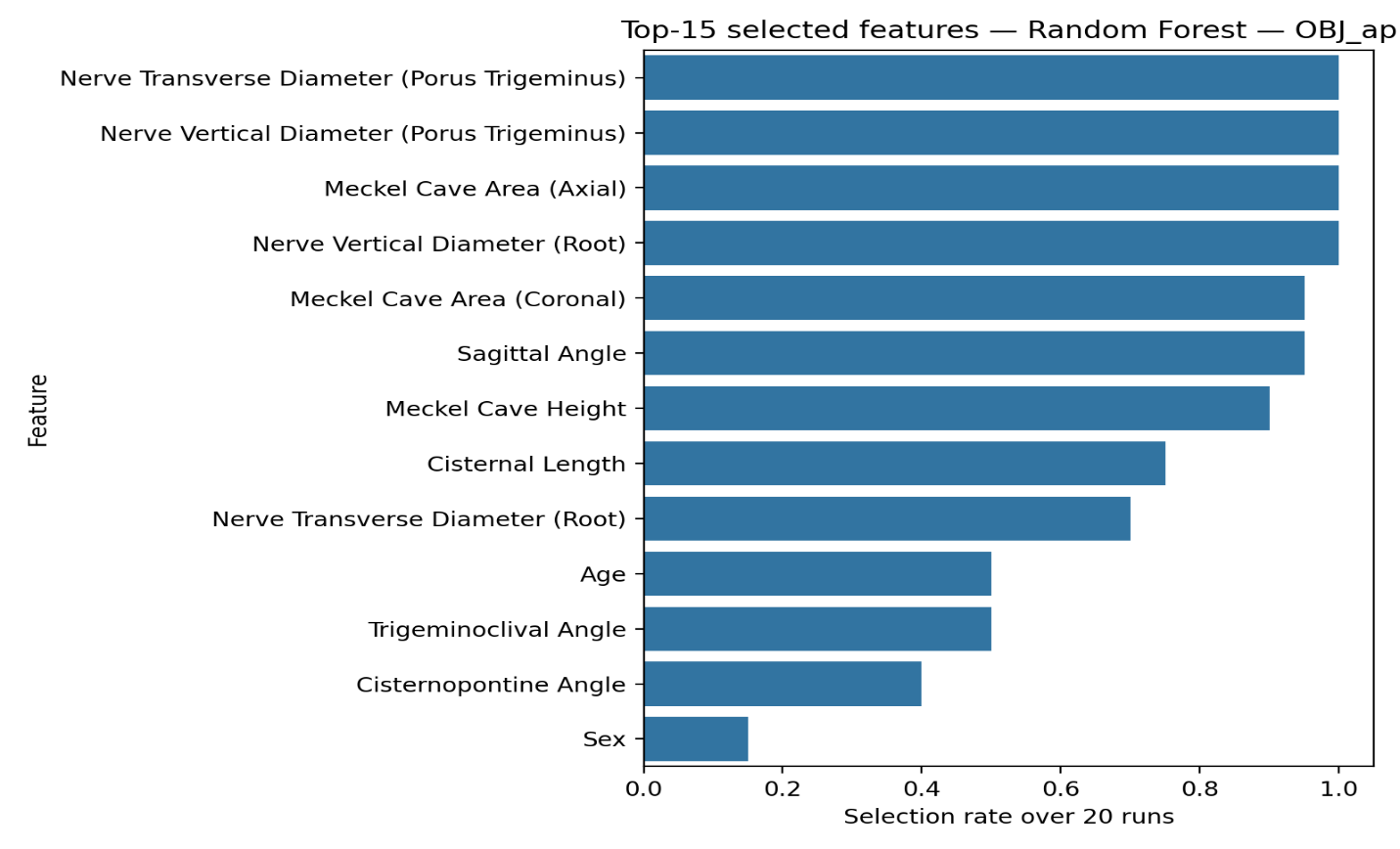

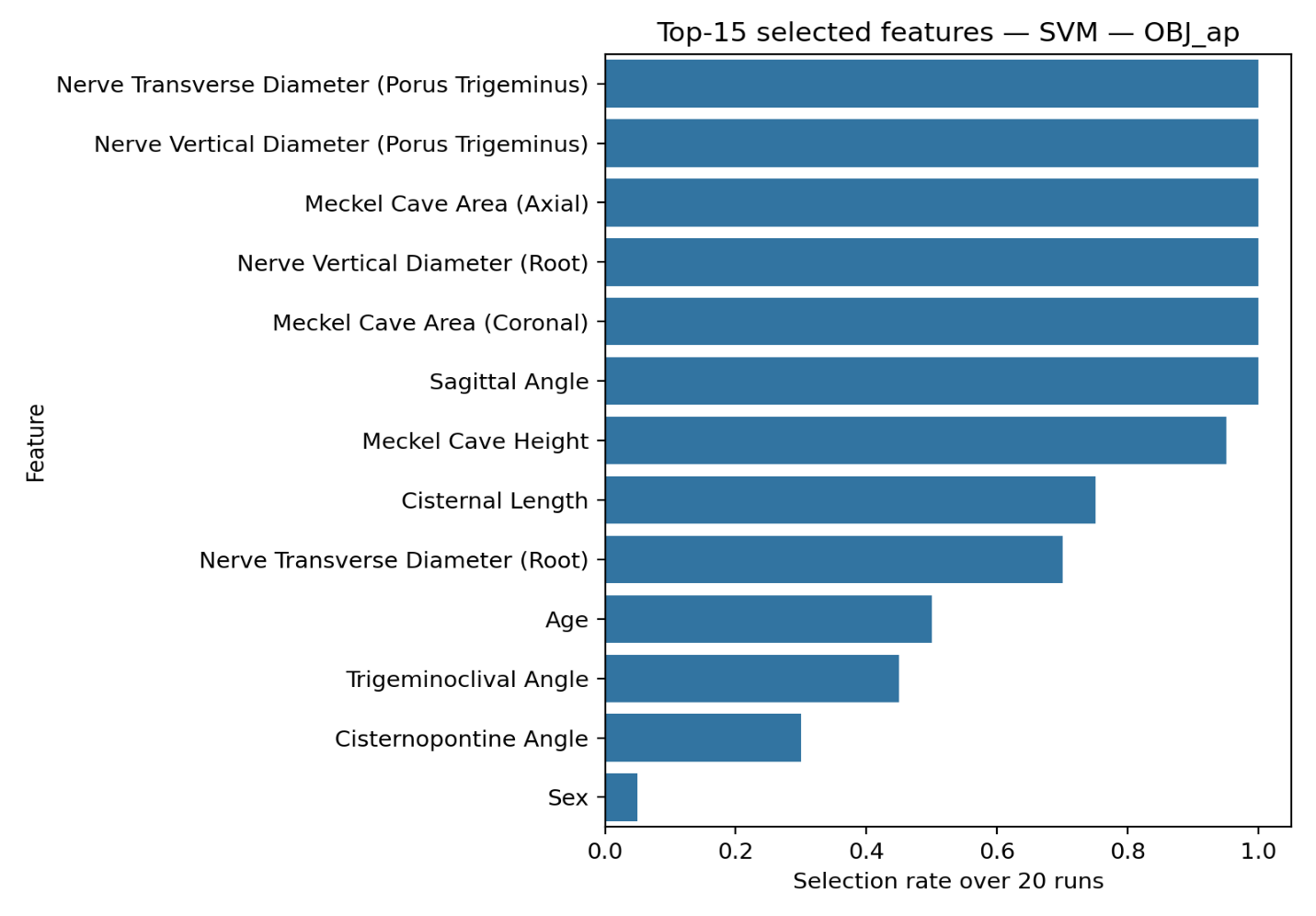


**Supplementary Table S4**. Mode and typical range (≈IQR) of best-trial hyperparameters across 20 outer repetitions

| **Model / Parameter** | **Mode (most frequent)** | **Typical range (≈IQR across best trials)** | **Notes** |
| --- | --- | --- | --- |
| **Random Forest** | | | |
| n_estimators | 300–500 | ~300–700 (200–1000 explored) | Medium–high forests common |
| max_depth | 12–16 | ~8–24 (2–30) | Moderate depth; avoids overfitting |
| min_samples_split | 2–6 | 2–10 |  |
| min_samples_leaf | 1–3 | 1–5 |  |
| max_features | sqrt / log2 | {sqrt,log2,None} | sqrt slightly most frequent |
| criterion | entropy / log_loss | {gini,entropy,log_loss} | non-Gini often selected |
| bootstrap | mixed (True/False) | — | No single dominant choice |
| **SVM** | | | |
| kernel | rbf (mode), also linear | {rbf, linear, poly} | poly rare (deg≈2–5) |
| C | 10–50 (mode band) | ~2–100+ (10th–90th) | Moderate–high penalties common |
| gamma (mode) | auto / scale | auto–scale–small float (1e-4–1e-2) | Few runs used explicit float γ |
| degree (if poly) | 3–4 | 2–5 | poly infrequent |
| probability | TRUE | — | for PR-AUC/ROC-AUC |
| **MLP** | | | |
| solver | lbfgs or adam | {lbfgs, adam} | both used across runs |
| hidden_layer_sizes | (20,10) / (20,10,5) / (15,15,5) | compact (2–3 layers) | 20–15–5 patterns recur |
| activation | relu (mode), tanh (2nd) | {relu, tanh, logistic} |  |
| alpha (L2) | ~1e-5–1e-3 | (1e-5–1e-3) | small weight decay |
| max_iter | ~700–1100 | 400–1200 | often converged before max |
| learning_rate (adam) | adaptive | {constant, adaptive} | init ~1e-4–1e-3; batch 16–64 |
| **KNN** | | | |
| n_neighbors | 6–14 | 3–25 |  |
| weights | distance (mode) | {distance, uniform} |  |
| metric / p | minkowski (p=1–2) | {minkowski, manhattan, euclidean} | p≈1–2 |
| leaf_size | ~15–40 | 10–60 | weak effect on best trials |
| **Bagging** | | | |
| base_estimator | tree (mode), also knn | {tree, knn} | tree base slightly dominant |
| n_estimators | 40–120 | 10–200 |  |
| max_samples | 0.6–0.9 | 0.5–1.0 |  |
| max_features | 0.6–1.0 | 0.5–1.0 |  |
| bootstrap / bootstrap_features | mixed | {True/False} | no single dominant setting |
| **XGBoost** | | | |
| n_estimators | 300–700 | 200–1000 |  |
| max_depth | 3–6 | 2–10 | shallow trees dominate |
| learning_rate | 0.05–0.20 | 0.01–0.30 | moderate η most common |
| subsample | 0.7–0.9 | 0.5–1.0 |  |
| colsample_bytree | 0.7–0.9 | 0.5–1.0 |  |
| min_child_weight | 1–3 | 0.5–5 |  |
| gamma | ~0–0.15 | 0–5 | mostly small |
| reg_alpha / reg_lambda | α≈0–0.01, λ≈0.5–2 | α: 0–0.5; λ: 0.5–5 | light–moderate regularization |
| scale_pos_weight | ~0.7–1.3× imbalance ratio | 0.5×–2× of neg/pos | tuned around class skew |

**Supplementary Figure S2.** *SHAP beeswarm plots for the five runs surrounding the median (median ± 2) across 20 repetitions.*


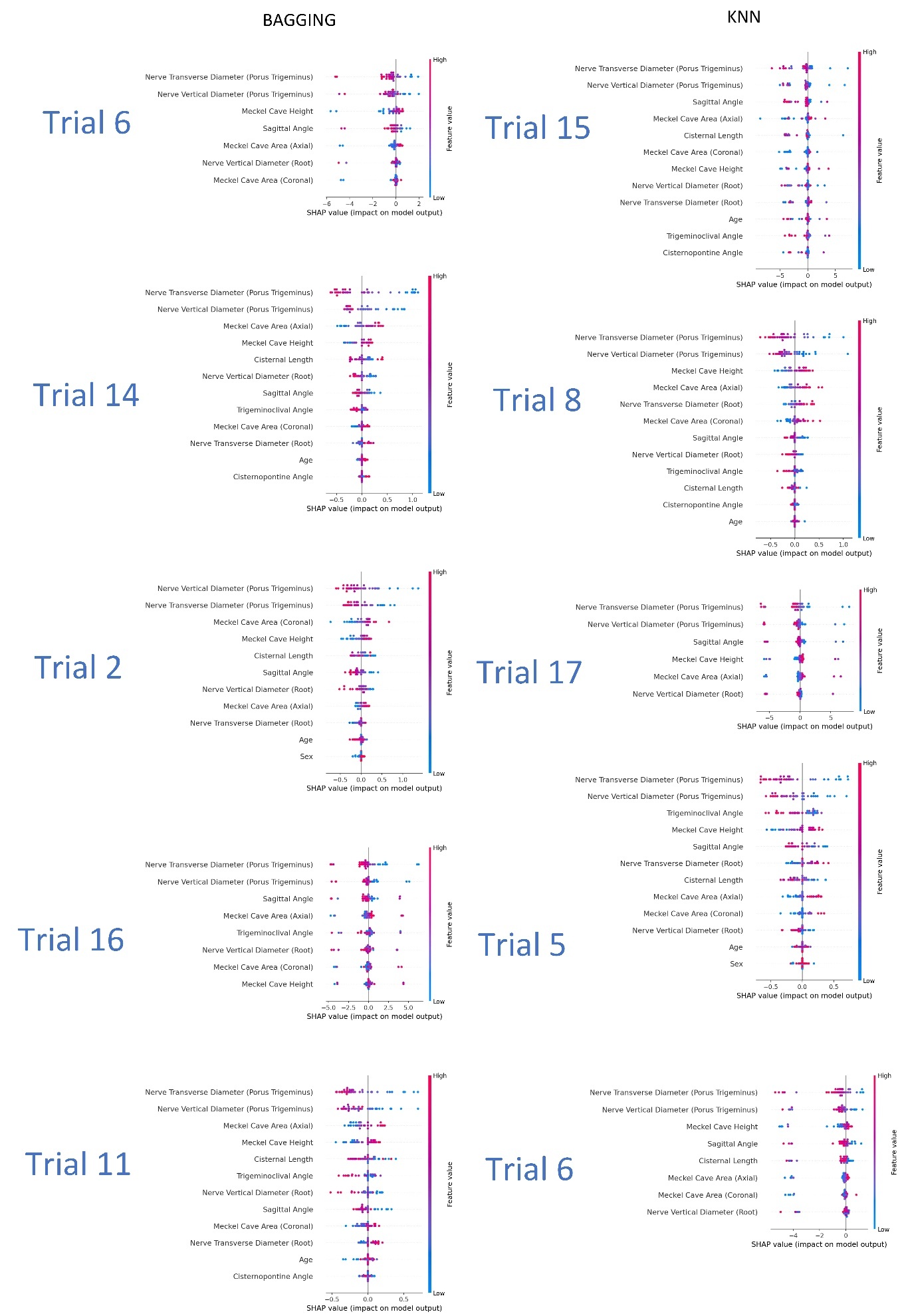


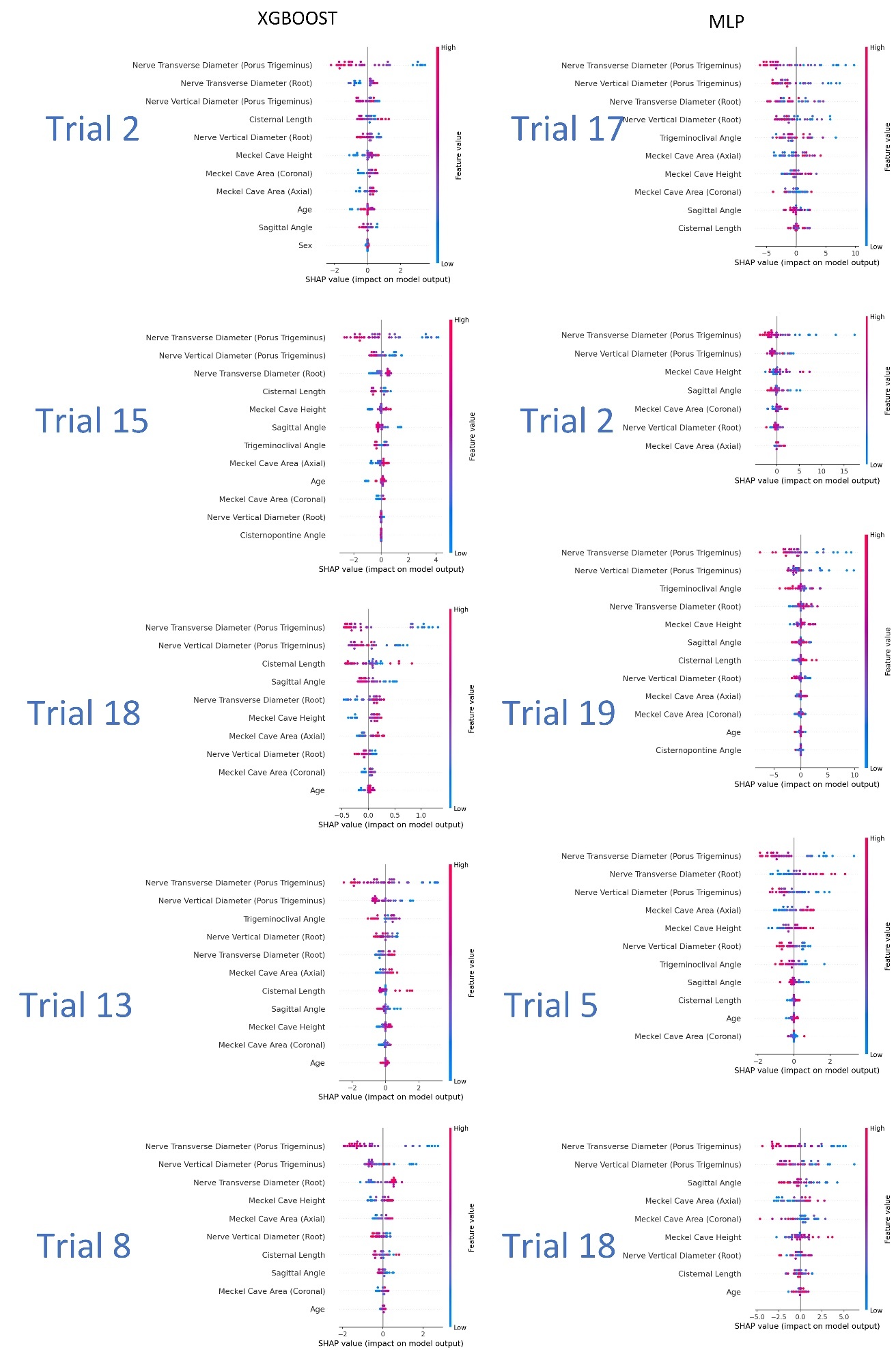


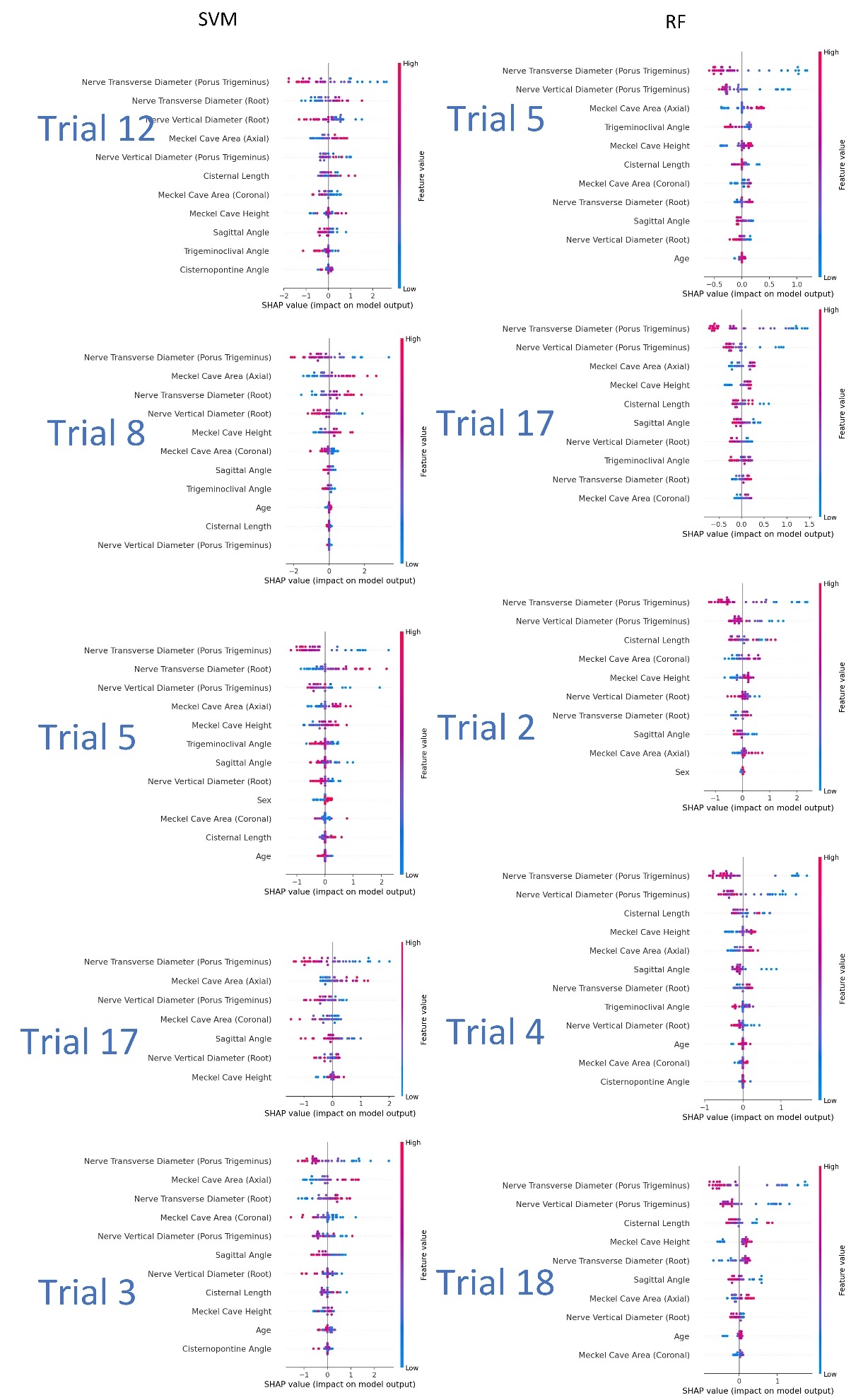


**Supplementary Figure S3.** *LIME explanations for the same test instance across the five runs surrounding the median (median ± 2).*


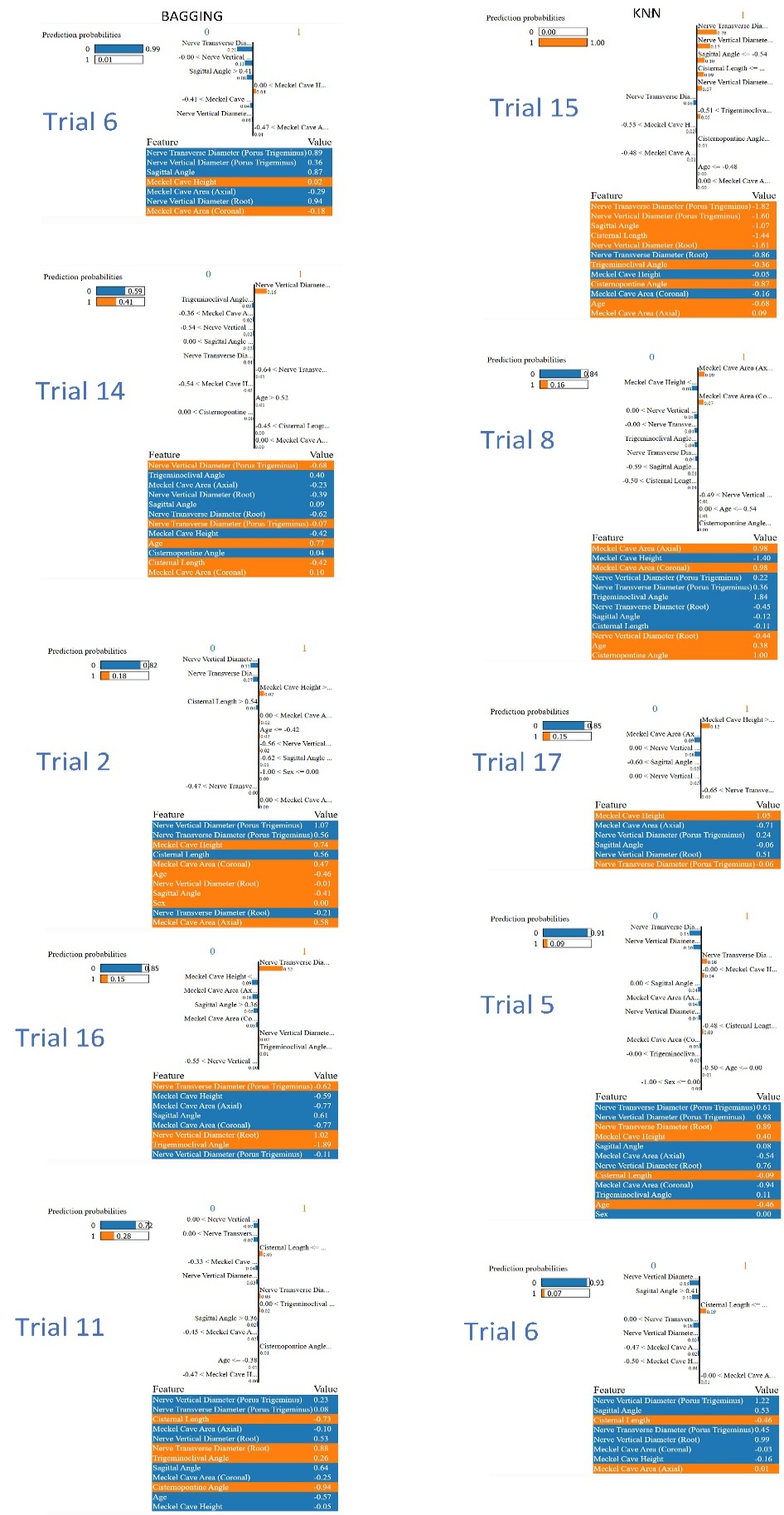


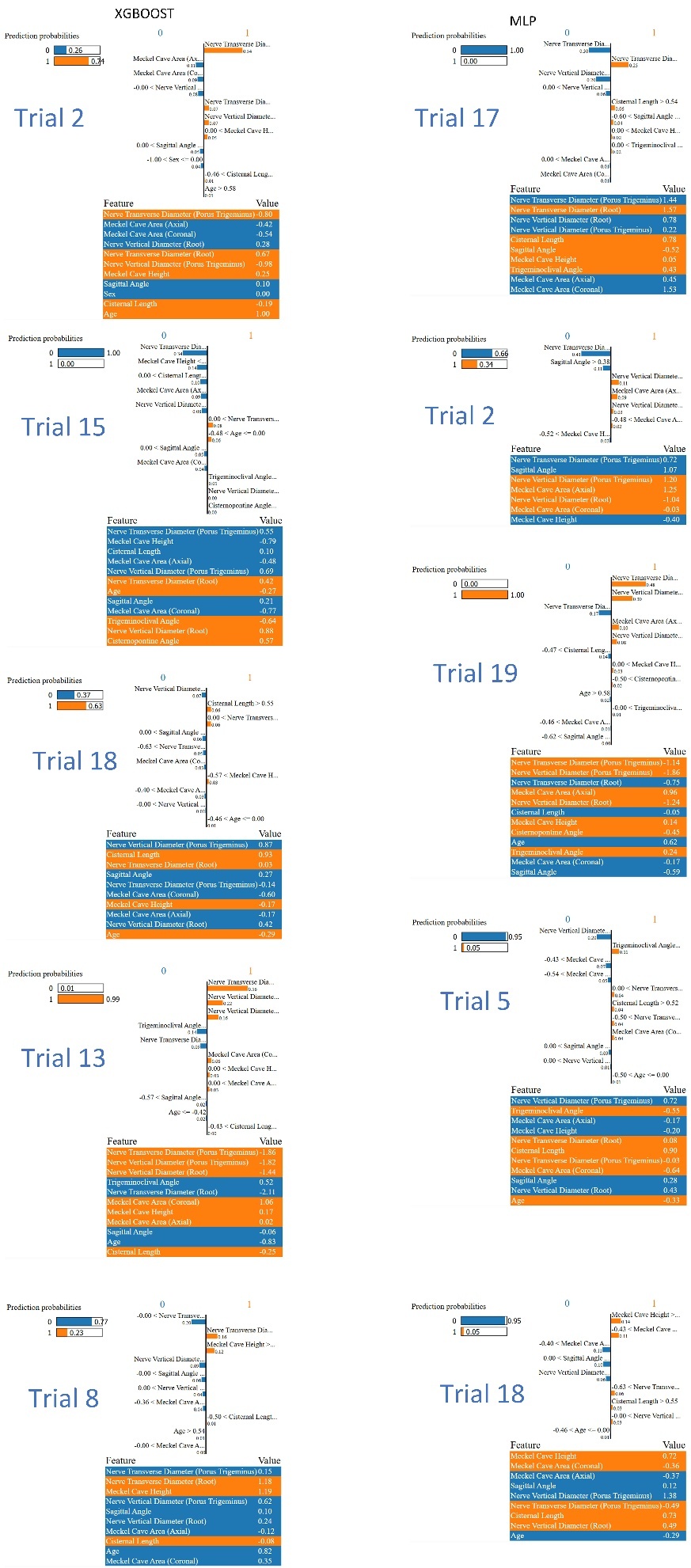


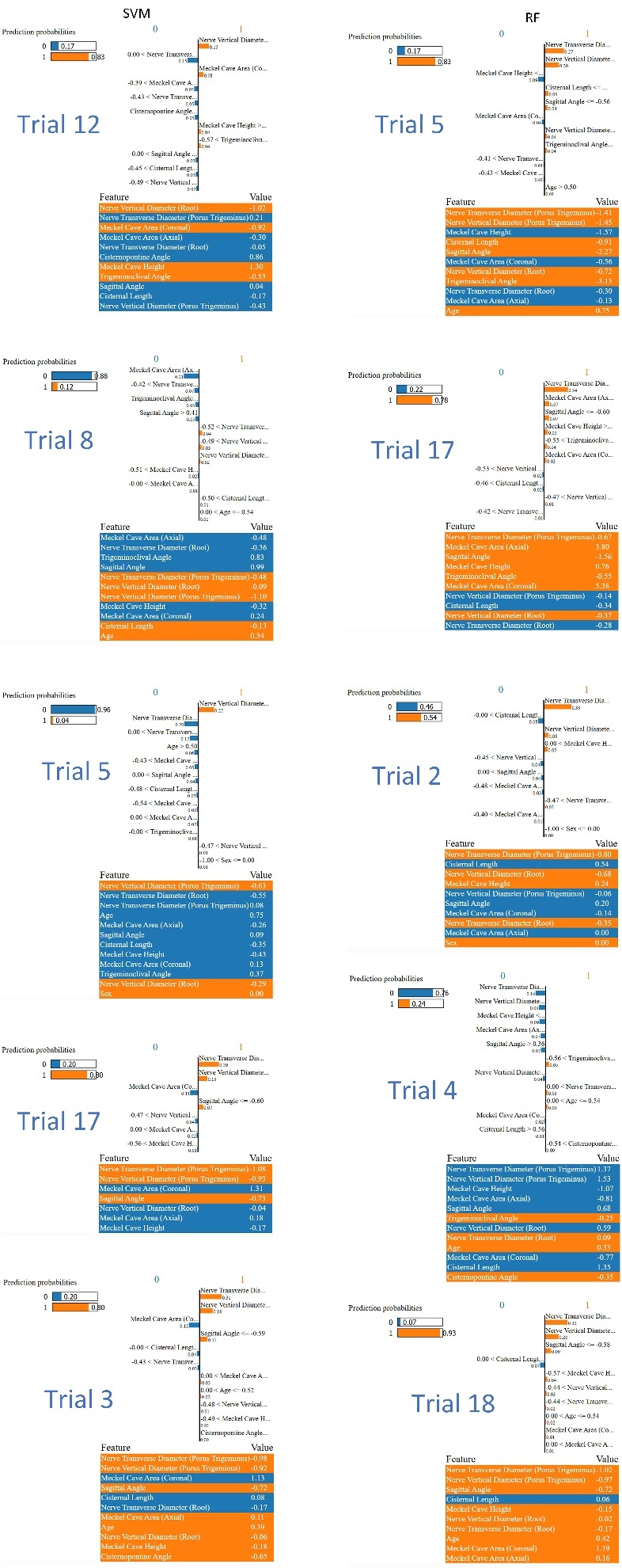

Supplement: Supplementary file 1 [file Supplementary_file_1.docx]
